# Protein Kinase C-delta (PKCδ) in Neurodegeneration and Cerebral Ischemia: Molecular Mechanisms and Therapeutic Implications

**DOI:** 10.1007/s12013-026-02125-w

**Published:** 2026-08-24

**Authors:** Yi-Heng Hsieh, I-Yen Lee, You Lien, Lon-Fye Lye, Yen-Chuan Ou, Ding-I Yang

**Affiliations:** 1 Institute of Brain Science, National Yang Ming Chiao Tung University, Taipei, 112304 Taiwan; 2 Division of Urology, Department of Surgery, Tungs’ Taichung MetroHarbor Hospital, Taichung, 435403 Taiwan; 3 Department of Medical Research, Tungs’ Taichung MetroHarbor Hospital, Taichung, 435403 Taiwan; 4 Brain Research Center, National Yang Ming Chiao Tung University, Taipei, 112304 Taiwan

**Keywords:** Apoptosis, Mitochondrial dysfunction, Neuroinflammation, Neuronal cell cycle reentry, Oxidative stress, Protein kinase C delta (PKCδ)

## Abstract

Protein kinase C delta (PKCδ), a calcium-independent novel PKC isoform, is increasingly recognized as a compartmentalized regulator of stress signaling in the central nervous system. Through tyrosine phosphorylation, subcellular translocation, regulation of its expression, and caspase-3-dependent proteolytic activation, PKCδ can convert transient adaptive responses into sustained oxidative, mitochondrial, inflammatory, and nuclear apoptotic signaling. This review evaluates PKCδ across Parkinson’s disease (PD), Alzheimer’s disease (AD), Huntington’s disease (HD), and ischemic stroke, emphasizing disease- and cell type-specific mechanisms rather than a uniformly pathogenic role. In PD, PKCδ most consistently amplifies toxin- and α-synuclein-associated dopaminergic injury and glial inflammation, although human evidence remains limited. In AD, PKCδ may link amyloidogenic processing of amyloid precursor protein (APP), amyloid-beta peptide (Aβ)-induced neuronal stress/cell cycle reentry, and glial neuroinflammation in a context-dependent feed-forward circuit. In HD, PKCδ signaling may shift from early compensatory downregulation to stress-induced apoptotic reactivation. In ischemic stroke, the strongest evidence implicates PKCδ in reperfusion-associated oxidative and inflammatory injury, particularly through neutrophil and translocation-dependent mechanisms. Future therapeutic development will require isoform-selective, compartment-specific, and cell type-resolved PKCδ modulation.

## Introduction to PKCδ

### PKC Family and Structure of PKCδ

Protein kinase C (PKC) comprises a family of lipid-regulated serine/threonine kinases that couple receptor-driven phospholipase C (PLC) signaling to diverse cellular responses. Mammalian PKCs are generally classified as conventional PKCs (α, βI, βII, γ), which require diacylglycerol (DAG) and Ca^2+^; novel PKCs (δ, ε, η, θ), which respond to DAG but are Ca^2+^-independent; and atypical PKCs (ζ, λ, ι), which lack canonical DAG/Ca^2+^-binding requirements and are regulated mainly by phosphorylation and protein interactions [1, 2]. This classification is important because PKCδ, unlike many conventional PKCs, is particularly responsive to cellular stress, oxidative injury, tyrosine phosphorylation, and apoptotic cleavage rather than only transient membrane-associated second-messenger signaling.

**Fig. 1:**
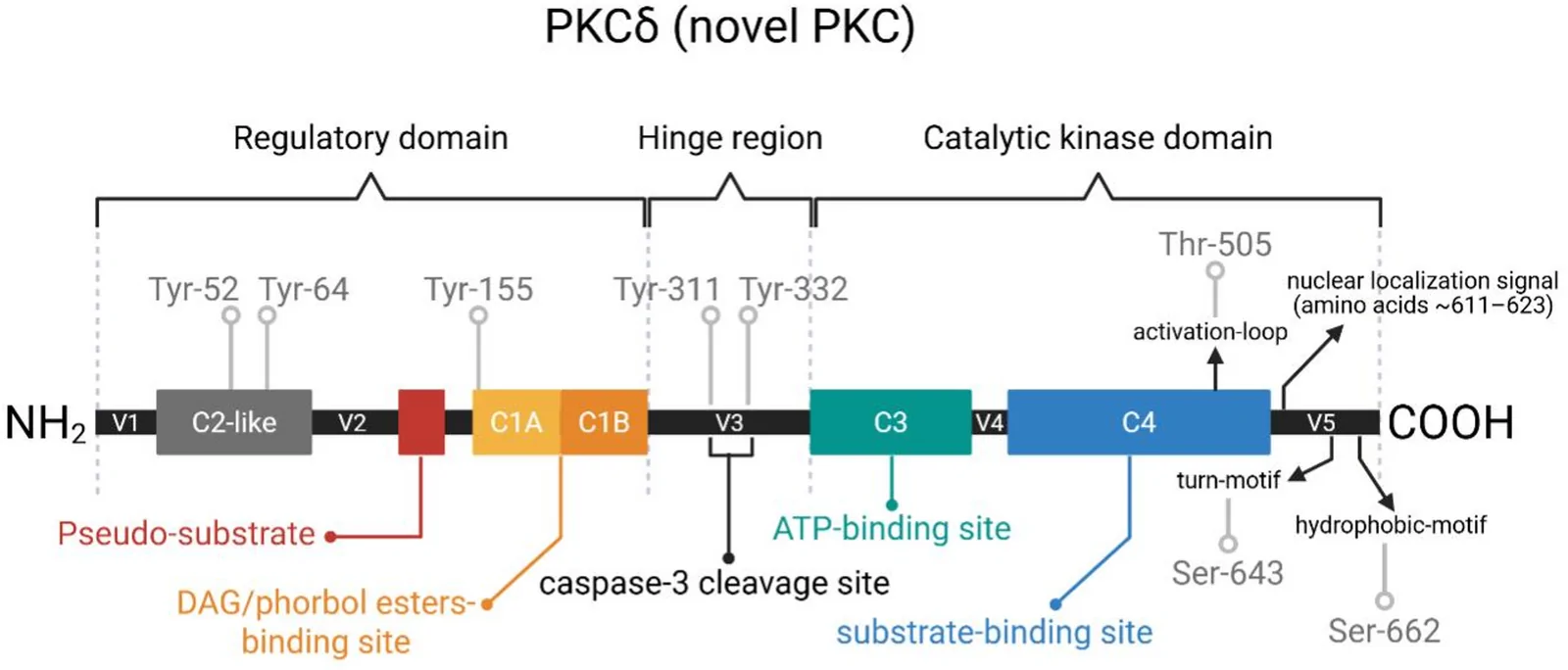
Domain architecture and regulatory features of PKCδ. Schematic representation of protein kinase C delta (PKCδ), a member of the novel PKC (nPKC) subfamily, showing its domain organization and major regulatory motifs. PKCδ contains an N-terminal regulatory domain, a hinge region, and a C-terminal catalytic kinase domain. The regulatory domain includes a C2-like domain, an autoinhibitory pseudosubstrate sequence, and tandem C1A/C1B domains that mediate DAG/phorbol ester binding. The hinge region contains the caspase-3 cleavage site, which separates the regulatory region from the catalytic fragment after proteolytic activation. The catalytic domain contains the C3 ATP-binding region and the C4 substrate-binding region. Regulatory phosphorylation sites shown include Tyr52, Tyr64, Tyr155, Tyr311, Tyr332, Thr505, Ser643, and Ser662, corresponding to residues discussed in the text. Thr505, Ser643, and Ser662 represent the activation-loop, turn-motif, and hydrophobic-motif priming sites, respectively. The C-terminal nuclear localization signal (NLS; amino acids ~ 611–623), conserved variable regions V1–V5, and key functional motifs are indicated. Human PKCδ residue numbering is shown

PKCδ, first identified in murine tissues in 1986, is a ubiquitously expressed novel PKC with a molecular weight of approximately 77.5 kDa [3]. It contains an N-terminal regulatory region with tandem C1 domains that bind DAG and phorbol esters, a C2-like domain that lacks conventional Ca^2+^-binding activity, a hinge/V3 region containing the caspase-3 cleavage site, and a C-terminal catalytic region with conserved phosphorylation sites, including Thr505, Ser643, and Ser662 (Fig. 1). These structural elements enable PKCδ to function not only as a lipid-sensitive kinase but also as a stress-activated effector whose localization, cleavage, and phosphorylation state strongly shape its biological output [2, 4, 5].

## Upstream Activation Mechanisms and Compartment-Specific Targeting of PKCδ

Activation of PKCδ is controlled through several interconnected mechanisms that include constitutive priming phosphorylation, lipid-dependent membrane recruitment, stress-responsive lipid-independent signaling, tyrosine phosphorylation, and caspase-3 cleavage.

### Priming Phosphorylation and Catalytic Maturation

Priming phosphorylation renders PKCδ catalytically competent before stimulus-dependent activation. Human PKCδ is phosphorylated at Thr505, Ser643, and Ser662, corresponding to Thr507, Ser645, and Ser664 in rodents. Unlike conventional PKCs, which are usually well phosphorylated at all three conserved sites, PKCδ often shows constitutive phosphorylation at Ser643 and Ser662 but relatively low basal phosphorylation at Thr505 [6, 7]. This pattern suggests that PKCδ relies heavily on context-dependent signals, especially subcellular targeting and stress-induced phosphorylation, to determine its downstream effects.

### Lipid-Dependent Membrane Recruitment and Activation

Canonical PKCδ activation begins when receptor-driven PLC signaling generates DAG within membrane microdomains. DAG recruits PKCδ to lipid bilayers through the tandem C1 domains, especially C1A, whereas the C1B domain shows higher affinity for phorbol esters such as phorbol 12-myristate 13-acetate (PMA; also known as 12-O-tetradecanoylphorbol-13-acetate or TPA) [6, 8–10]. Because PKCδ has a nonfunctional C2-like domain, membrane recruitment is Ca^2+^-independent and is influenced by ligand identity, membrane composition, and acidic phospholipids such as phosphatidylserine [11–13]. Thus, membrane translocation is not merely redistribution but a major determinant of PKCδ signaling specificity.

### Stress-Responsive Mitochondrial Targeting and Noncanonical Signaling

Beyond plasma-membrane signaling, PKCδ can translocate to mitochondria under oxidative stress, DNA damage, or ischemia/reperfusion, where it promotes mitochondrial dysfunction, cytochrome c release, and apoptotic commitment. Studies in keratinocytes, toxin-treated cells, and cardiac ischemia/reperfusion models show that mitochondrial PKCδ contributes to loss of mitochondrial membrane potential (MMP), BCL2-associated agonist of cell death (BAD)-associated injury, reactive oxygen species (ROS) amplification, and caspase activation [14–18]. These findings support the concept that stress-responsive mitochondrial targeting converts upstream injury signals into a pro-death signaling program [12, 19, 20].

Within the mitochondrial intermembrane space, PKCδ can also be activated through a DAG-independent redox mechanism involving the p66Shc/cytochrome c/retinol signalosome. In this complex, phosphorylated Tyr332 of mouse PKCδ binds the SH2 domain of p66Shc, while cytochrome c is positioned on the same p66Shc platform to promote site-specific oxidation of the PKCδ RING-finger-like cysteine-rich C1 domains, with retinol serving as an electron-transfer catalyst [21].

### Tyrosine Phosphorylation, Caspase-3 Cleavage, and Nuclear Accumulation

PKCδ is also regulated by lipid-independent mechanisms involving tyrosine phosphorylation, nuclear translocation, and caspase-3-mediated cleavage. Under oxidative stress, DNA damage, and other pro-apoptotic conditions, Src family kinases and related tyrosine kinases phosphorylate PKCδ at multiple residues, thereby altering conformation, protein interactions, and localization [5, 12, 22]. Tyr52 promotes interaction with SH2-domain proteins such as Lyn, whereas Tyr64 and Tyr155 can expose a cryptic C-terminal nuclear localization signal (NLS) and facilitate importin-α-dependent nuclear entry [23, 24]. Thus, individual tyrosine sites control distinct signaling outputs rather than representing a uniform activation marker.

Tyrosine phosphorylation in the hinge region plays another distinct role in coupling PKCδ to proteolytic activation. In particular, Tyr332 is directly implicated in promoting efficient caspase-3-dependent cleavage; Tyr311, which lies adjacent to the caspase cleavage site, has also been implicated in this priming process, but with less direct mechanistic evidence [12, 25]. Caspase-3-mediated cleavage removes the N-terminal regulatory moiety of PKCδ and generates a constitutively active catalytic fragment [26, 27]. This proteolytic event functionally reinforces apoptotic signaling and favors nuclear accumulation of the catalytic fragment through the C-terminal NLS [28].

Nuclear PKCδ is a key effector of apoptosis and can regulate targets including signal transducer and activator of transcription-1 (STAT1)- and p53-dependent pathways [29–31]. Nuclear translocation and caspase activation may reinforce each other in a feed-forward loop, converting reversible stress signaling into commitment to cell death [20, 28, 32]. Consistently, blockade of Tyr64/Tyr155 phosphorylation protects against radiation-induced salivary gland injury, and c-Abl-mediated Tyr311 phosphorylation can activate p38 and apoptosis in glioma cells [33, 34].

## Downstream Compartment-Specific Pathogenic Signaling of PKCδ

**Fig. 2:**
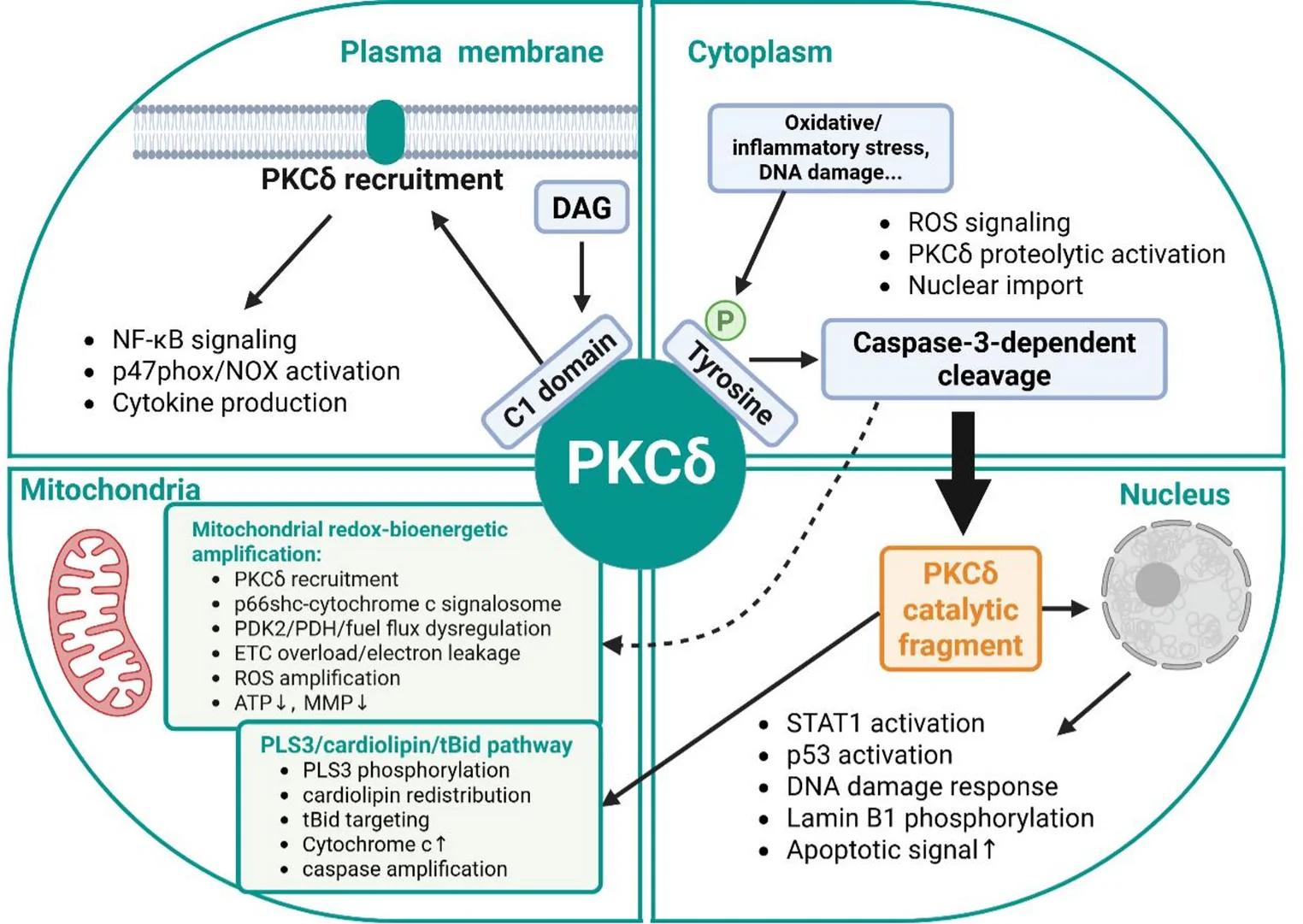
Shared compartment-specific pathogenic mechanisms of PKCδ signaling. PKCδ signaling is shaped by activation mode and subcellular localization. At the plasma membrane, DAG-dependent C1-domain recruitment links PKCδ to NF-κB signaling, p47phox/NOX activation, and cytokine production. In the cytoplasm, oxidative or inflammatory stress and DNA damage promote tyrosine phosphorylation, which functions mainly as a routing or priming mechanism by regulating ROS signaling, subcellular targeting, nuclear import, and susceptibility to proteolytic cleavage. In contrast, caspase-3-dependent cleavage generates a constitutively active PKCδ catalytic fragment, favoring commitment and amplification of cell-death signaling. Mitochondrial PKCδ contributes to redox-bioenergetic amplification through p66Shc–cytochrome c–retinol-associated signaling, PDK2/PDH-linked fuel-flux dysregulation, electron leakage, ROS amplification, ATP depletion, and MMP loss. It also promotes PLS3/cardiolipin/tBid-dependent cytochrome c release and caspase amplification. Nuclear PKCδ supports STAT1- and p53-associated apoptotic transcription, DNA damage responses, Lamin B1 phosphorylation, and nuclear execution

The preceding sections establish how PKCδ is activated through phosphorylation, proteolytic cleavage, and stimulus-dependent targeting to distinct subcellular compartments. This section extends that framework by focusing on what these compartmentalized PKCδ pools do after activation, particularly how they amplify oxidative stress, mitochondrial dysfunction, nuclear apoptotic signaling, and inflammatory injury across cellular stress paradigms relevant to central nervous system (CNS) injury. At different subcellular compartments, membrane-, mitochondrial-, cytoplasmic-, and nuclear-associated PKCδ pools produce overlapping but distinguishable injury outputs, which are summarized as shared pathogenic mechanisms in Fig. 2.

### Mitochondrial Redox-bioenergetic Amplification

PKCδ interaction with mitochondria appears to involve an isozyme-specific recruitment and retention mechanism that differs from canonical DAG/phorbol ester-dependent translocation to the plasma membrane or Golgi. Live-cell fluorescence resonance energy transfer (FRET) imaging showed that PKCδ can interact with, and remain active at, the mitochondrial outer membrane through determinants involving its C1 domains, turn motif, C2-like domain, catalytic activity, and MMP [35]. Once localized to mitochondria, PKCδ may amplify ROS by altering respiratory and redox control. In particular, the mitochondrial PKCδ/retinol signal complex, involving p66Shc, cytochrome c, and retinol, has been proposed to regulate pyruvate dehydrogenase kinase-2/ pyruvate dehydrogenase (PDK2/PDH)-dependent fuel flux and to monitor cytochrome c redox state in the intermembrane space [21]. Physiologically, this signalosome may tune glucose-derived substrate entry into the Krebs cycle. Under sustained stress, however, prolonged PKCδ-linked respiratory activation may overload the electron transport chain, thereby increasing electron leakage, enhancing ROS formation, and causing mitochondrial dysfunction. This model links mitochondrial PKCδ localization to energy imbalance and ROS amplification.

### Mitochondrial Lipid Remodeling, tBid Targeting, Cytochrome c Release, and Caspase Amplification

A second mitochondrial mechanism links PKCδ to intrinsic apoptosis through lipid remodeling. Phospholipid scramblase 3 (PLS3) was identified as a mitochondrial target of PKCδ-induced apoptosis; PKCδ physically interacts with and phosphorylates PLS3, and mitochondrial-targeted PKCδ enhances PLS3-dependent apoptosis [36]. Mechanistically, phosphorylation of PLS3 at Thr21 increases lipid-scrambling activity, promotes cardiolipin redistribution to the mitochondrial surface, facilitates mitochondrial targeting of truncated Bid (tBid), and enhances apoptotic signaling [37]. Endoplasmic reticulum (ER) stress can also transmit apoptotic signals to mitochondria through a PKCδ-Abl complex, supporting an inter-organelle stress-propagation model [38]. This pathway explains how mitochondrial PKCδ can sensitize mitochondria to Bcl-2 family-mediated permeabilization, cytochrome c release, caspase-9/caspase-3 activation, and subsequent apoptotic amplification. A related downstream logic applies to ischemic neuronal injury, where PKCδ/Drp1-associated fission is linked to loss of MMP, reduced ATP, ROS, and apoptosis [39].

### Nuclear Apoptotic and Transcriptional Execution

Nuclear PKCδ represents a functionally distinct pool. Whereas mitochondrial PKCδ mainly amplifies redox, bioenergetic, and intrinsic apoptotic injury, nuclear PKCδ couples stress signaling to transcriptional and structural death execution. The catalytic fragment generated by caspase-3 cleavage contains a C-terminal NLS and can accumulate in the nucleus, where PKCδ has been linked to STAT1-dependent [29] and p53-dependent [40, 41] apoptotic signaling, DNA damage responses [42, 43], and phosphorylation of nuclear substrates such as lamin B1 [44]. Thus, nuclear PKCδ should not be treated as simply a relocated form of mitochondrial PKCδ. Rather, it may convert upstream oxidative or genotoxic stress into apoptotic gene expression, lamina disruption, and death commitment. This distinction is relevant to dopaminergic toxin models, p53-linked HD mechanisms, and neuronal stress pathways.

### Membrane-Associated NOX/NF-κB/Inflammatory Signaling

Beyond mitochondria and the nucleus, membrane- and cytoplasm-associated PKCδ pools can amplify inflammatory and oxidant signaling. At plasma membrane or particulate compartments, PKCδ can interact with oxidase-associated proteins, including p47phox, thereby promoting NADPH oxidase (NOX)-dependent ROS generation in reperfusion or inflammatory settings [45, 46]. In microglia and astrocytes, PKCδ also contributes to nuclear factor-kappaB (NF-κB) activation, cytokine production, inflammasome priming, and chemotactic or cytoskeletal responses [47–54]. This arm is important because extramitochondrial ROS and cytokines can secondarily impair mitochondrial function in neurons and glia [45, 47–51, 54]. Therefore, the mitochondrial-redox loop and membrane-associated inflammatory loop are not independent; they can reinforce each other through ROS, proinflammatory cytokines like tumor necrosis factor-α (TNF-α), interleukin-1β (IL-1β), and other stress mediators.

### Tyrosine Phosphorylation versus Caspase Cleavage: Routing/Priming versus Commitment/Amplification

Tyrosine phosphorylation and caspase cleavage should be interpreted as related but non-equivalent regulatory modes. Tyrosine phosphorylation often functions as a routing or priming signal: it can modify PKCδ conformation, protein interactions, targeting, nuclear import, and susceptibility to proteolytic cleavage. In contrast, caspase-3 cleavage removes the N-terminal regulatory domain and generates a constitutively active catalytic fragment, favoring sustained kinase activity and apoptotic commitment [55]. This distinction helps reconcile disease-specific patterns. In acute or toxin-associated neuronal injury, caspase-3-dependent PKCδ cleavage is frequently linked to irreversible apoptotic amplification, whereas in chronic CNS disorders, tyrosine phosphorylation, expression-level induction, or recruitment may dominate earlier stress responses. Importantly, these modes are not mutually exclusive: phosphorylation can facilitate cleavage or localization, whereas cleavage can redistribute active PKCδ to mitochondria or nucleus. Thus, compartment, activation mode, and cell type determine whether PKCδ acts primarily as a redox amplifier, mitochondrial apoptotic effector, inflammatory mediator, or nuclear execution kinase.

## PKCδ Expression and Disease-Relevant Activation in the CNS

Having outlined the general mechanisms of PKCδ activation, compartmental targeting, and downstream pathogenic signaling, the next question is how these regulatory events are reflected in CNS-relevant experimental systems. In the non-neuronal tumor cells under genotoxic stress, it has been reported that p53 induces PKCδ transcription, and PKCδ may in turn reinforce p53 expression and activity, thereby forming a slower positive feedback pathway that sustains apoptotic signaling after acute post-translational activation has begun [42, 56, 57]. In neuronal systems, oxidative stress is the best-established trigger of PKCδ activation. In dopaminergic models, oxidative insults induce tyrosine phosphorylation and caspase-3-dependent cleavage of PKCδ, producing a catalytic fragment that promotes apoptosis [58–60]. Oxidative stress can also increase Prkcd transcription through mechanisms involving long isoform Sp3, cAMP response element-binding protein (CREB), and p300, linking acute activation to delayed expression-level reinforcement [61]. Outside dopaminergic paradigms, PKCδ has been implicated in motor neuron disease, seizure-induced hippocampal apoptosis, status epilepticus-associated inflammation, migraine-related signaling, traumatic brain injury, and sepsis-associated damage of blood-brain barrier (BBB) [62–67]. However, the functional meaning of PKCδ activation differs across models, and direct mechanistic evidence remains comparatively limited in Alzheimer’s disease (AD) and Huntington’s disease (HD). The current literature therefore supports a strong role for PKCδ in selected oxidative stress-driven neuronal injuries, especially dopaminergic degeneration, but does not justify a uniform pathogenic role across all CNS disorders.

## Rationale and Aims

**Fig. 3:**
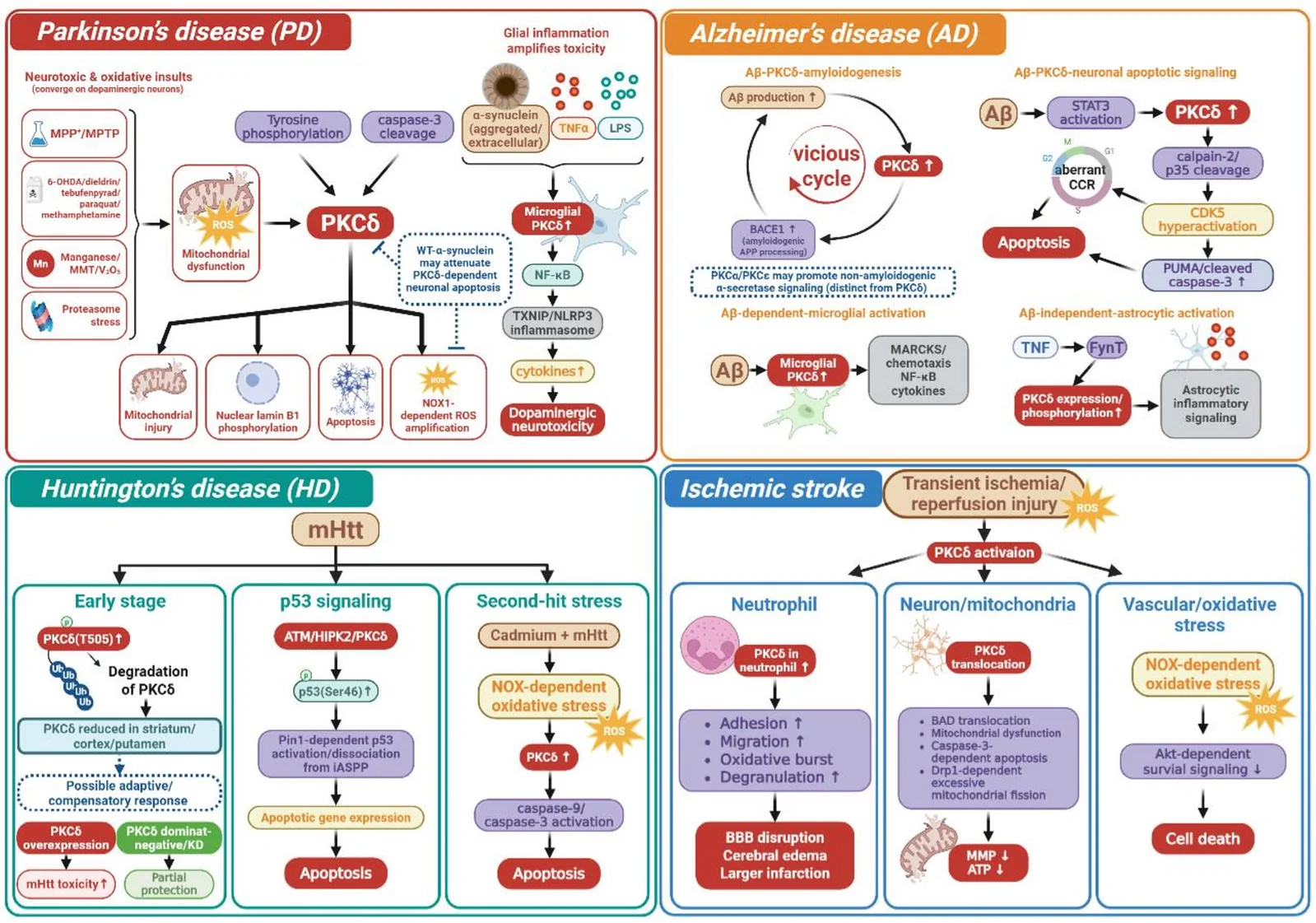
Disease-specific pathogenic mechanisms of PKCδ across major CNS disorders. PKCδ contributes to CNS pathology through disease- and cell type-specific signaling modules rather than a uniform pathogenic program. In PD, neurotoxic and oxidative insults converge on mitochondrial dysfunction, tyrosine phosphorylation, and caspase-3-dependent PKCδ activation in dopaminergic neurons, promoting mitochondrial injury, nuclear lamin B1 phosphorylation, ROS amplification, and neuronal apoptosis. Microglial PKCδ further amplifies α-synuclein-, TNF-α-, and LPS-associated neuroinflammatory signaling through NF-κB activation, TXNIP/NLRP3 inflammasome priming, and cytokine production. In AD, PKCδ participates in an Aβ–PKCδ–BACE1/amyloidogenic APP-processing vicious cycle, Aβ-induced neuronal cell cycle reentry (CCR) and apoptosis, as well as Aβ-dependent or Aβ-independent glial inflammatory responses. In HD, PKCδ appears context-, stage-, and stress-dependent, with early reduction possibly representing an adaptive response, whereas PKCδ activation contributes to p53-mediated apoptosis and second-hit oxidative injury. In ischemic stroke, reperfusion-associated PKCδ activation promotes neutrophil-mediated inflammation, mitochondrial apoptotic signaling, vascular oxidative injury, BBB disruption, edema, and infarct expansion. Solid arrows indicate experimentally supported relationships, whereas dashed annotations denote context-dependent or inferential links

Several reviews have addressed PKCδ in apoptosis, DNA damage, broad disease biology, or dopaminergic neurotoxicity [20, 32, 43, 68]. However, an updated disease-integrated assessment across adult CNS disorders remains lacking. This review therefore focuses on PKCδ in PD, AD, HD, and ischemic stroke by distinguishing shared compartment-specific injury mechanisms from disease- and cell-type-specific signaling contexts, while also evaluating therapeutic modulation and translational limitations. Disease- and cell type-specific PKCδ signaling modules across these disorders are summarized in Fig. 3.

## Parkinson’s Disease

PD is characterized by progressive degeneration of substantia nigra pars compacta dopaminergic neurons, striatal dopamine depletion, and motor impairment [69]. Although multifactorial, PD pathogenesis repeatedly involves mitochondrial dysfunction, oxidative stress, impaired proteostasis, and chronic neuroinflammation [70]. PKCδ is positioned at the intersection of these processes because it is activated by oxidative stress, tyrosine phosphorylation, caspase-3 cleavage, and compartment-specific translocation. Evidence from toxin-based, inflammatory, and genetic models therefore supports PKCδ as a mechanistic amplifier, not merely a marker, of dopaminergic neurodegeneration [12, 20, 71].

### Neurotoxin-Induced Proteolytic Activation of PKCδ in Dopaminergic Neuronal Death and Synaptic Degeneration

Experimental links between PKCδ and PD emerged most clearly from neurotoxin models. 1-Methyl-4-phenylpyridinium (MPP^+^), the active metabolite of 1-methyl-4-phenyl-1,2,3,6-tetrahydropyridine (MPTP), disrupts fast axonal transport through caspase- and PKCδ-dependent mechanisms and induces oxidative stress, mitochondrial dysfunction, cytochrome c release, caspase-3 activation, and PKCδ cleavage in dopaminergic cells [72–75]. Importantly, PKCδ RNA interference or cleavage-resistant PKCδ mutants attenuate MPP^+^-induced tyrosine hydroxylase (TH)-positive neuronal degeneration, supporting a causal role for proteolytic PKCδ activation [76].

Similar convergence is seen with dieldrin, 6-hydroxydopamine (6-OHDA), and tebufenpyrad. These toxicants promote mitochondrial injury, oxidative stress, caspase-3 activation, and PKCδ cleavage in dopaminergic models; in the case of tebufenpyrad, activated PKCδ also translocates to the nucleus and phosphorylates lamin B1, linking PKCδ to nuclear lamina disruption [44, 77–81]. These studies place PKCδ downstream of mitochondrial injury but upstream of sustained apoptotic and nuclear execution programs.

Metal-associated neurotoxicity further extends the pathogenic relevance of PKCδ in parkinsonian degeneration. For example, caspase-3-dependent proteolytic activation of PKCδ plays a key role in manganese-induced apoptotic cell death in dopaminergic neuronal cells [82]. Exposure to methylcyclopentadienyl manganese tricarbonyl (MMT) elicits dose- and time-dependent increases in ROS, mitochondrial cytochrome c release, caspase-3 activation, DNA fragmentation, and sustained PKCδ cleavage in dopaminergic neurons [83]. Welding fume-associated vanadium pentoxide similarly promotes proteolytic conversion of native PKCδ into an active 41-kDa catalytic fragment [84]. Chronic manganese exposure, although more commonly associated with manganism than classical idiopathic PD, is also relevant because it activates PKCδ-dependent stress signaling to disrupt neuron-glia coupling. In astrocytes, manganese-dependent proteolytic activation of PKCδ impairs expression of glutamine transporter and glutamine uptake, thereby disrupting metabolic coupling between neurons and astrocytes, thereby indirectly exacerbating dopaminergic vulnerability [85].

Together, across structurally diverse toxicants, PKCδ emerges as a recurrent stress-activated kinase whose proteolytic conversion links mitochondrial injury to amplification of apoptotic and degenerative signaling in dopaminergic neurons.

### PKCδ as a Redox-Sensitive Amplifier of Oxidative Stress

The toxin literature converges on oxidative stress, a central pathogenic feature of PD. ROS generated by mitochondrial poisons, dopamine oxidation, paraquat, methamphetamine, and other insults can activate PKCδ directly or indirectly through tyrosine kinases and caspase-3 [12, 22, 60]. PKCδ is also a feed-forward amplifier: in paraquat-exposed dopaminergic neurons, it upregulates NOX1 to enhance ROS production, and in methamphetamine (MA) models, pharmacological inhibition or genetic deletion of PKCδ attenuates oxidative injury and behavioral deficits [86, 87]. Similar PKCδ-dependent redox toxicity has been reported after androgen exposure and in paraquat-treated microglia [88, 89].

### PKCδ in Neuroinflammatory Signaling and Glia-Mediated Neurotoxicity

PKCδ also contributes to glia-mediated neurotoxicity in PD. In dopaminergic neurons, TNF-α induces caspase-8/3-dependent PKCδ cleavage, nuclear translocation, and apoptosis, whereas TNF neutralization or PKCδ deletion is protective [90]. In microglia, α-synuclein, TNF-α, and lipopolysaccharide (LPS) increase PKCδ expression and kinase activity, promoting inflammatory mediator release and dopaminergic toxicity [47]. Mechanistically, α-synuclein activates Toll-like receptor 2 (TLR2)/cluster of differentiation-36 (CD36)-Fyn-PKCδ signaling, leading to NF-κB activation and priming of inflammasome components such as pro-IL-1β and NLRP3; PKCδ also links α-synuclein-induced ER stress to TXNIP-dependent inflammasome activation [49, 50, 91]. MPTP-induced activation of astrocytes involves tumor necrosis factor-like weak inducer of apoptosis (TWEAK)-dependent induction of PKCδ and STAT3/NOD-like receptor family CARD domain-containing protein 4 (NLRC4) signaling with concomitant loss of TH-positive neurons [51].

### Context-Dependent Modulation of PKCδ by α-Synuclein

The relationship between PKCδ and α-synuclein is bidirectional and context-dependent. In some neuronal paradigms, wild-type α-synuclein interacts with PKCδ and BAD or suppresses p300/NF-κB-dependent PKCδ transcription, thereby attenuating downstream apoptosis [61, 92–94]. In contrast, aggregated or extracellular α-synuclein can drive Fyn-PKCδ-dependent microglial inflammation and inflammasome signaling [49, 91]. Thus, α-synuclein does not act in a simple linear pathway with PKCδ; rather, its effect depends on cell type, stress context, aggregation conditions, and disease stage.

### Translational Relevance and Limitations of Toxin-Based PKCδ Models

The mechanistic evidence linking PKCδ to dopaminergic degeneration is strongest in neurotoxin- and stress-based paradigms, including MPP^+^/MPTP, 6-OHDA, paraquat, methamphetamine, manganese, dieldrin, tebufenpyrad, and proteasome-inhibition models. These systems repeatedly converge on mitochondrial dysfunction, oxidative stress, caspase-3 activation, PKCδ cleavage or phosphorylation, and apoptotic or inflammatory amplification [44, 72–87]. Thus, they provide persuasive mechanistic support for PKCδ as a stress-responsive amplifier of dopaminergic injury. However, they should not be interpreted as complete models of idiopathic PD. Many toxin paradigms produce relatively acute dopaminergic injury and do not fully reproduce the chronic, multifactorial, α-synuclein-associated progression of sporadic disease [75]. Therefore, toxin-induced PKCδ signaling is best viewed as modeling convergent injury modules shared with PD, rather than establishing PKCδ as an initiating driver of human idiopathic PD.

Direct human evidence also remains limited and should be interpreted with caution. First, available human transcriptomic studies provide strong evidence for mitochondrial, proteostatic, inflammatory, and cell-type-specific alterations in PD substantia nigra, but they do not yet establish *PRKCD* as a consistently dysregulated transcript across idiopathic PD datasets. Human proteomic datasets support the relevance of mitochondrial and inflammatory pathways that can converge on PKCδ signaling, but unbiased proteomics has not yet clearly established PKCδ itself as a dominant differentially abundant protein in sporadic PD substantia nigra. Direct phosphoproteomic evidence also remains limited, but targeted postmortem immunoblotting/immunohistochemistry now supports increased PKCδ activation in PD substantia nigra. Thus, increased phospho-PKCδ Thr505 and downstream Lamin B1 phosphorylation or loss were reported in postmortem substantia nigra tissues from PD patients, providing targeted evidence that PKCδ activation may also occur in vulnerable nigral dopaminergic neurons [44]. Another postmortem study reported upregulation of PKCδ in microglia within the ventral midbrain of PD patients, supporting disease relevance of microglial PKCδ signaling [47]. Nevertheless, these clinical findings are based on a small number of targeted postmortem analyses, and they do not establish whether PKCδ activation occurs early, late, or as a compensatory response during disease progression. In α-synuclein preformed-fibril models, activation of the PKCδ-ER stress-thioredoxin-interacting protein (TXNIP)/NLR family pyrin domain-containing 3 (NLRP3) axis precedes delayed TH-positive neuronal loss, suggesting that microglial PKCδ may participate relatively early in α-synuclein-driven inflammation [50]. By contrast, proteolytic or nuclear PKCδ activation in toxin-treated dopaminergic neurons appears more consistent with a downstream amplifier of oxidative, mitochondrial, and caspase-dependent injury [44, 58–60, 72–81]. Overall, available data support PKCδ as a disease-relevant preclinical signaling node in PD, but additional cell type-specific human studies and activation-state measurements are required before PKCδ can be regarded as a clinically validated therapeutic target.

### Therapeutic Strategies by PKCδ Modulations for PD

Mounting evidence identifies PKCδ as an important mediator of dopaminergic neurodegeneration and, therefore, a plausible candidate therapeutic target in PD models (Table 1). Pharmacological blockade of caspase-3-mediated PKCδ cleavage with Z-Asp(OMe)-Ile-Pro-Asp(OMe)-FMK (z-DIPD-fmk) completely rescued TH-positive neurons from MPP⁺- and 6-OHDA-induced toxicity in mouse primary mesencephalic cultures [95]. In parallel, rottlerin was reported to protect PC12 cells against 6-OHDA cytotoxicity while suppressing PKCδ phosphorylation [96]. However, although these findings support the concept that PKCδ inhibition is beneficial, the mechanistic interpretation is not equally robust for all agents because rottlerin is now recognized as a non-selective modulator rather than a definitive PKCδ-specific inhibitor.

**Table 1:** Pharmacological and experimental strategies targeting PKCδ in PD

Compound/Tool	Study Model	Downstream Effect	Mechanism	Ref.
• PKCδ siRNA • cleavage-resistant PKCδ^D327A^ mutant	• dopaminergic cell culture • Wistar rat (MA toxin models)	• MA-induced autophagy as a protective strategy for inhibiting mitochondria-mediated apoptosis and degradation of aggregated proteins	• reduced MA-induced autophagy, UPS dysfunction, ubiquitin aggregates, and cell death	[99]
• PKCδ siRNA • catalytically inactive PKCδ^K376R^ mutant • cleavage-resistant PKCδ^D327A^ mutant • z-DEVD-fmk*	• N27 cells • primary mouse mesencephalic cultures • C57 mouse (6-OHDA toxin models)	• PKCδ^D327A^, PKCδ^K376R^ or PKCδ siRNA protected against 6-OHDA-induced N27 cell death • PKCδ^D327A^ protected primary mouse mesencephalic cultures from 6-OHDA-induced apoptosis • PKCδ^D327A^ protected against 6-OHDA-induced PKCδ activation in mouse substantia nigra	• z-DEVD-fmk irreversibly inhibited caspase-3 cleavage of PKCδ • blocked 6-OHDA-induced DNA fragmentation	[81]
• PKCδ^-/-^ mice • rottlerin*	• WT and PKCδ^-/-^ mice (MA toxin models)	• rottlerin attenuated behavioral impairments, oxidative stress, and TUNEL^+^-apoptotic cells in WT mice • MA-induced behavioral impairments were not apparent in PKCδ^-/-^ mice	• MA increased PKCδ expression in the striatum • MA-induced increases in the dopamine turnover rate, decreases in TH activity, as well as expression of TH, DAT, and VMAT2 were not observed in rottlerin-treated WT or PKCδ^-/-^ mice	[87]
• rottlerin* • PMA*	• PC12 cells (6-OHDA toxin model)	• rottlerin protected PC12 cells from 6-OHDA toxicity • PMA strengthened 6-OHDA toxicity	• rottlerin and PMA respectively inhibited and enhanced PKCδ phosphorylation	[96]
• z-DIPD-fmk* • z-DEVD-fmk*	• N27 cells • primary mouse mesencephalic cultures (MPP^+^/6-OHDA toxin models)	• attenuated cytotoxicity in N27 cells • rescued TH^+^ neurons in primary mouse mesencephalic cultures	• blocked activation of caspase-3, but not caspase-9 • blocked PKCδ cleavage, proteolytic activation of PARP, and DNA fragmentation	[95]
• z-DIPD-fmk*	• N27 cells exposed to H_2_O_2_ (a chronic low-dose oxidative stress model)	• protected against H_2_O_2_-induced apoptosis	• blocked H_2_O_2_-induced caspase-3 activation and PKCδ cleavage	[60]
• paeoniflorin*	• PC12 cells (6-OHDA toxin model)	• suppressed mitochondria-mediated apoptosis • enhanced antioxidant capability by increasing glutathione	• attenuated 6-OHDA-induced NF-κB translocation without affecting phosphorylation of Akt, JNK, p38, and ERK1/2 • blocked PKCδ upregulation • ↓ ROS/PKCδ/NF-κB signaling pathway	[97]
• paeoniflorin*	• MPTP-treated mouse	• restored the motor performance impairment caused by MPTP • inhibited apoptosis and protected the ultrastructure of neurons	• ↑ anti-apoptotic Bcl-2 • ↓ pro-apoptotic Bax, α-synuclein, and PKCδ • ↓ phosphorylated active form of NF-κB p65 subunit	[98]

Bax, Bcl2-associated X; Bcl-2, B-cell lymphoma-2; DAT, dopamine transporter; ERK1/2, extracellular signal-regulated kinase-1/2; JNK, c-Jun N-terminal kinase; MA, methamphetamine; MPP^+^, 1-methyl-4-phenylpyridinium; MPTP, 1-methyl-4-phenyl-1, 2, 3, 6-tetrahydropyridine; NF-κB, nuclear factor-kappaB; N27, rat mesencephalic dopaminergic neuronal cell line; 6-OHDA, 6-hydroxydopamine; PARP, poly-(ADP-ribose) polymerase; paeoniflorin, an active compound of Radix Paeoniae Alba; PC12, rat pheochromocytoma cell line; PMA, phorbol 12-myristate 13-acetate; rottlerin, mallotoxin, a polyphenol product isolated from *Mallotus philippensis*; ROS, reactive oxygen species; TH, tyrosine hydroxylase; TUNEL, terminal deoxynucleotidyl transferase-mediated dUTP nick-end labeling; UPS, ubiquitin-proteasome system; VMAT2, vesicular monoamine transporter-2; WT, wild-type; z-DIPD-fmk, an irreversible peptide inhibitor for caspase-3 cleavage of PKCδ; z-DEVD-fmk, caspase-3 inhibitor. *: non-specific for PKCδ

Natural compounds have also shown neuroprotective potential in paradigms that converge on PKCδ signaling. Paeoniflorin, a bioactive constituent of Radix Paeoniae Alba (white peony root, derived from *Paeonia lactiflora* Pall.), attenuated 6-OHDA-induced apoptosis in PC12 cells through inhibition of the ROS-dependent PKCδ/NF-κB pathway [97]. In vivo, paeoniflorin increased anti-apoptotic signaling, including upregulation of Bcl-2, while reducing PKCδ and α-synuclein expression at both the mRNA and protein levels, thereby conferring protection in the MPTP mouse model of PD [98]. Consistent with these observations, z-DIPD-fmk also markedly protected against oxidative stress-induced apoptotic death, further underscoring the pathological relevance of proteolytic PKCδ activation [60]. Taken together, these data strengthen the view that limiting stress-driven PKCδ activation can interrupt convergent apoptotic pathways in dopaminergic neurons.

Psychostimulant-induced neurotoxicity provides additional mechanistic insight into PKCδ-dependent degeneration. MA markedly increased PKCδ expression in the striatum, whereas genetic deletion of PKCδ or pharmacological treatment with rottlerin attenuated behavioral deficits and oxidative injury, including lipid peroxidation and protein oxidation [87]. MA exposure also induced autophagy together with impaired proteasomal function, increased PKCδ and caspase-3 activation, and accumulation of ubiquitinated aggregates and LC3-II. Importantly, PKCδ knockdown or expression of a cleavage-resistant mutant mitigated these pathological changes and improved cell survival, indicating that sustained PKCδ activation contributes to ubiquitin-proteasome system dysfunction and caspase-3-dependent apoptosis within the nigrostriatal pathway [99]. Moreover, MA-induced PKCδ upregulation impaired TH phosphorylation at Ser40 and produced behavioral abnormalities without changing dopamine content or total TH expression; these effects were reversed by rottlerin treatment or PKCδ knockout [100]. Overall, the genetic evidence is particularly persuasive in supporting a causal contribution of PKCδ to toxin-associated dopaminergic injury.

Proteasome impairment represents another upstream trigger of PKCδ activation. Exposure to the proteasome inhibitor MG-132 induced caspase-3-dependent PKCδ cleavage and promoted mitochondrial translocation of the catalytic fragment. In this setting, inhibition of the caspase-3/PKCδ axis prevented activation of caspase-9 and caspase-3, suggesting that mitochondrial accumulation of active PKCδ amplifies the intrinsic apoptotic cascade [101]. Collectively, the PD literature supports a model in which proteolytically activated PKCδ participates in a feed-forward loop linking oxidative stress, proteasome dysfunction, mitochondrial injury, and apoptosis. At the same time, the translational strength of the field is limited by heavy reliance on toxin-based models and by the incomplete selectivity of some pharmacological tools, highlighting the need for more isoform-selective PKCδ inhibitors and validation in disease-relevant in vivo systems.

One widely used pharmacological inhibitor of PKCδ is rottlerin; however, accumulating evidence clearly demonstrates that its biological effects cannot be attributed to selective inhibition of this kinase. Thus, rottlerin may best be considered as a non-selective modulator of PKCδ rather than a specific PKCδ inhibitor. In primary astrocytes, rottlerin rapidly inhibits Na⁺-dependent glutamate transport activity and markedly reduces glutamate-aspartate transporter (GLAST) immunoreactivity within 5 min, suggesting accelerated GLAST degradation or limited proteolysis [102]. Although phorbol esters acutely activate PKC isoforms, chronic phorbol ester exposure is commonly used to downregulate PKCδ through sustained activation followed by depletion. Notably, in primary astrocytes, such PKCδ downregulation failed to prevent rottlerin-mediated inhibition of glutamate transport activity, supporting a PKCδ-independent effect of rottlerin on GLAST regulation [102].

Mechanistically, rottlerin functions predominantly as a mitochondrial uncoupler. It disrupts MMP, reduces intracellular ATP levels, activates AMP-activated protein kinase (AMPK), and alters ROS production. These metabolic disturbances can indirectly suppress PKCδ activation, particularly by limiting ATP-dependent phosphorylation events, thereby mimicking kinase inhibition. In addition, rottlerin exhibits broad off-target activity, including modulation of multiple kinases and ion channels, especially Ca²⁺-activated K⁺ channels, which further complicates interpretation of experimental outcomes [103]. Recent studies reinforce this lack of specificity. For instance, in neutrophils, rottlerin fails to inhibit phorbol ester-induced ROS production, whereas the selective PKCδ inhibitory peptide KAI-9803 (delcasertib) effectively suppresses this response [104]. Collectively, these findings indicate that rottlerin-mediated effects should not be interpreted as evidence of PKCδ inhibition. Continued reliance on rottlerin as a PKCδ-selective inhibitor is therefore inappropriate and may lead to misleading conclusions.

### Integrative Perspective for Therapeutic Implications

Overall, PD provides the strongest neurodegenerative evidence for PKCδ as a pathogenic but context-dependent hub. In neurons, PKCδ links mitochondrial dysfunction and oxidative stress to caspase-dependent apoptosis; in glia, it amplifies NF-κB- and inflammasome-related inflammatory signaling. The concept is supported across toxicants, oxidants, cytokines, α-synuclein, and genetic models, but translation remains limited by heavy reliance on toxin paradigms, incomplete cell type mapping, and non-selective pharmacological tools [15, 17, 20, 28, 47].

## Alzheimer’s Disease

AD is the leading cause of dementia and is pathologically defined by extracellular deposition of amyloid-beta (Aβ) peptides, intracellular neurofibrillary tangles composed of hyperphosphorylated tau, synaptic degeneration, and progressive neuronal loss [105, 106]. Although the “amyloid cascade hypothesis” has been refined over time, abnormal amyloid precursor protein (APP) processing and accumulation of toxic Aβ assemblies remain central to most mechanistic models of AD [107].

Against this background, PKCδ deserves attention because it is a stress-responsive, Ca^2+^-independent PKC isoform positioned at the intersection of oxidative signaling, apoptosis, inflammatory activation, and in selected contexts of amyloidogenic APP processing. Notably, PKCδ can be framed as a cell-type-specific feed-forward pathogenic amplifier in AD by acting on multiple steps including APP processing, neuronal stress, or glial inflammation. In neurons, Aβ-induced PKCδ expression/activation may drive aberrant cell cycle reentry and apoptotic signaling while also converging with established PKCδ-associated stress pathways such as mitochondrial dysfunction, oxidative stress, ROS amplification, and caspase-dependent injury. Reciprocally, heightened PKCδ can further promote β-site APP cleaving enzyme-1 (BACE1)-dependent amyloidogenic APP processing, thereby increasing Aβ generation and reinforcing an Aβ–PKCδ–BACE1 vicious cycle. Increased neuronal amyloidogenic burden may then provide a persistent stimulus for microglial and astrocytic activation, whereas PKCδ within glial cells can further amplify Aβ-dependent and Aβ-independent neuroinflammatory signaling through NF-κB-related cytokine production and sustained inflammatory activation. Thus, neuronal and glial PKCδ pathways may converge to link amyloidogenesis, neuronal injury, and inflammatory amplification into a cohesive pathogenic circuit.

### Human Evidence and Interpretive Limitations

Evidence from human tissue remains limited and somewhat conflicting. An earlier study reported that Ca^2+^-independent PKC activity and PKCδ protein levels in cytosolic and membrane fractions were not significantly altered in postmortem AD cortex relative to controls [108]. By contrast, a more recent study found increased expression of PKCδ and BACE1 in AD brain and a positive correlation between the two proteins [109]. These findings should not be treated as directly equivalent because they differed in cortical region analyzed, tissue handling, biochemical fractionation strategy, and likely postmortem interval, which may substantially influence preservation of kinase activity and apparent subcellular distribution. Moreover, total protein abundance may be less informative than activation state, phosphorylation pattern, cleavage status, or cell-type-specific localization. Thus, the current human data are compatible with PKCδ involvement in AD, but do not yet establish whether PKCδ is an initiating driver, a secondary stress response, or a stage-specific modifier or even amplifier of pathology in AD patients. These observations from human brain tissues therefore support a cautious, mechanism-specific interpretation rather than a simple conclusion that PKCδ is uniformly increased or invariably pathogenic in AD.

### Amyloidogenic APP Processing: Distinguishing PKCδ from PKCα/PKCε

Different PKC isoforms appear to play differential roles in APP processing. PKCδ has been shown to enhance Aβ production via promotion of β-secretase-associated APP processing. Increased PKCδ levels positively correlate with BACE1 expression in the postmortem AD brain, suggesting a potential association between PKCδ and amyloidogenic APP processing; consistently, PKCδ knockdown reduces BACE1 expression, BACE1-mediated APP processing, and Aβ production [109]. Pharmacological inhibition of PKCδ by rottlerin further reduces Aβ production and improves cognitive performance in APPswe/PS1dE9 mice, supporting a functional link between PKCδ activity, amyloid burden, and cognitive impairment in vivo [109]. Likewise, lysophosphatidic acid (LPA), the major bioactive component of oxidized low-density lipoprotein (LDL), enhances Aβ generation by increasing BACE1 expression through Tyr311 phosphorylation of PKCδ [110]. These observations support a pro-amyloidogenic role for PKCδ under stress-related conditions.

In contrast to the pro-amyloidogenic role for PKCδ, classical APP-processing studies indicate that PKCα and PKCε, but not PKCδ, more consistently promote the non-amyloidogenic α-secretase pathway [111, 112]. An earlier study showed that, in rat 3Y1 fibroblasts stably overexpressing three different PKC isoforms, the levels of secreted APP were higher in cells overexpressing PKCα or PKCε than in PKCδ-transfected cells or vector-only controls [111]. In CHO cells that express human APP751, expression of the selective peptide inhibitor of PKCε inhibited PMA-induced APPs secretion with suppression of Aβ production; in contrast, the PKCδ peptide inhibitor had no effect, suggesting that PKCε decreases Aβ production by promoting α-secretase-mediated cleavage of APP [113]. Later, another study also reported that PKCα and PKCε, but not PKCδ, promote non-amyloidogenic α-secretase cleavage with significantly increased sAPPα secretion in HEK293 or rhabdomyosarcoma cells [112]. Taken together, these findings appear to suggest that PKCδ is more closely linked to amyloidogenic signaling and BACE1 upregulation, whereas PKCα and PKCε drive α-secretase-dependent nonamyloidogenic APP processing.

Presenilin-1/-2 (PS1/2) is the catalytic subunit of γ-secretase complex involved in APP processing [114, 115]. Expression of various PKC isoforms was evaluated in the mouse embryonic fibroblasts (MEFs) lacking either PS1, PS2, or both [116]. Results revealed differential regulation of PKC isoforms by PS1/2: reduced levels of PKCα and PKCγ were observed in PS1/2 double-knockout MEFs, whereas PKCδ levels were significantly elevated in both PS1/2 double-knockout and PS1-deficient MEFs and were lowered upon re-expression of PS1. While these findings suggest that PS-dependent signaling can selectively modulate expression of different PKC isoforms, the use of MEFs rather than disease-relevant neural cells limits the interpretation of these results in the context of AD pathophysiology.

### Neuronal Stress, Cell Cycle Reentry, and Apoptosis

Besides promoting amyloidogenic APP processing to enhance Aβ production by PKCδ, exogenous Aβs may conversely trigger expression of PKCδ to form a vicious cycle. For example, both Aβ25–35 and Aβ1–42 induce PKCδ expression in primary rat cortical neurons [117]. Aberrant neuronal cell cycle reentry is increasingly recognized as an important mechanism in AD, linking mitogenic stress to apoptotic death rather than successful division [118, 119]. Consistently, suppression of Aβ25–35/1-42-induced PKCδ preserves cell viability and neuronal morphology while attenuating expression of cell cycle markers like cyclin D1, proliferating cell nuclear antigen (PCNA), and phospho-histone H3 induction in the fully differentiated post-mitotic cortical neurons [117]. Mechanistically, signal transducer and activator of transcription-3 (STAT3) lies upstream of PKCδ, whereas calpain-2-mediated p35 cleavage, hyperactivation of cyclin-dependent kinase-5 (CDK5), induction of p53-upregulated modulator of apoptosis (PUMA), and caspase-3 cleavage lie downstream, placing PKCδ within a plausible Aβ-driven death cascade [117]. This pathway is attractive because it connects Aβ exposure to a well-recognized but still under-integrated feature of AD biology, namely cell cycle dysregulation in vulnerable neurons [120]. Caution is needed in judging the generalizability of this mechanism because cultured neuronal systems cannot fully model the chronic, multicellular environment of the diseased brain. Nonetheless, given the involvements of PKCδ in mitochondrial dysfunction, oxidative stress and apoptosis, this body of work strongly supports PKCδ as a mediator of Aβ-induced neuronal stress responses, at least partially, in addition to acting on amyloidogenic APP processing for enhanced Aβ production. Taken together, Aβ-induced PKCδ may contribute to multiple neuronal stress responses, including mitochondrial dysfunction, oxidative stress, neuronal cell cycle reentry, and apoptosis. Together with PKCδ-mediated enhancement of Aβ production, these processes may form a vicious cycle that amplifies Aβ- and PKCδ-dependent neuronal death in AD.

### Glial Neuroinflammation and PKCδ-Dependent Inflammatory Amplification

In glial neuroinflammation, PKCδ may amplify sustained inflammatory responses at two different tiers, namely Aβ-dependent and Aβ-independent mechanisms. Microglia and astrocytes actively shape disease progression by modulating cytokine production, phagocytosis, synaptic remodeling, and responses to Aβ and tau pathology in AD [121, 122]. In microglia, Aβ selectively activates PKCδ, driving phosphorylation and translocation of myristoylated alanine-rich C kinase substrate (MARCKS), membrane-cytoskeletal remodeling, and chemotaxis [53]. More recent work further indicates that PKCδ is elevated in cerebrospinal fluid of AD patients and positively correlated with neuroinflammation markers; moreover, PKCδ expression is detected in activated microglia of AD mouse brains, where it facilitates NF-κB signaling and cytokine overproduction; genetic or pharmacological suppression of PKCδ attenuates microglial activation while improving cognition in AD-related models [54]. Besides Aβ-dependent PKCδ activation, the pro-inflammatory cytokine TNF alone without Aβ is sufficient to induce FynT in astrocytes to promote late-phase PKCδ expression and phosphorylation, thereby constituting an Aβ-independent inflammatory mechanism [52]. These two PKCδ-dependent mechanisms may be interconnected, forming a feed-forward inflammatory circuit that sustains and amplifies neuroinflammation in AD.

In addition to direct involvements in glial neuroinflammation, modulation of PKCδ also indirectly contributes to the anti-inflammatory effects of various reagents. For example, acetylpuerarin reduces microglial activation and lowers PKCδ-associated inflammatory markers in vivo and in BV2 cells [123, 124]. Naringenin also suppresses inflammatory gene expression partly through a suppressor of cytokine signaling-3 (SOCS3) pathway linked to PKCδ [125, 126]. Likewise, ethanolamine plasmalogens blunt PKCδ-associated inflammatory signaling in murine models [127]. Overall, these studies place PKCδ within pro-inflammatory glial response pathways, although its relative contribution may vary according to glial cell type, disease stage, and experimental intervention.

### Therapeutic Modulation and the Isoform-Specificity Problem

Therapeutic interpretation of PKCδ in AD should follow the pathogenic-amplifier model outlined above. If PKCδ contributes to amyloidogenic Aβ generation, Aβ-induced neuronal stress/apoptotic vulnerability, and glial inflammatory amplification, then PKCδ suppression could in principle interrupt several convergent pathological processes rather than a single isolated pathway. This possibility is supported by experimental strategies summarized in Table 2: rottlerin and genetic suppression of PKCδ reduce BACE1 expression, BACE1-mediated APP processing, and Aβ production, while PKCδ shRNA attenuates Aβ-induced neuronal cell cycle reentry and downstream apoptotic signaling [109, 117]. However, these findings should be interpreted as preclinical and mechanism-specific evidence rather than proof that PKCδ is a clinically validated AD target.

**Table 2:** Pharmacological and experimental strategies targeting PKCδ in AD

Compound /Tool	Study Model	Downstream Effect	Mechanism	Ref.
• PKCδ shRNA	• fully differentiated primary rat cortical neuron treated with Aβ	• suppression of Aβ-induced cell cycle reentry in post-mitotic neurons and subsequent neuronal apoptosis	• STAT3-dependent PKCδ induction triggers calpain2-mediated p35 cleavage into p25 to overactivate CDK5, thus causing aberrant cell cycle reentry, PUMA induction, and caspase-3 cleavage	[117]
• overexpression of PKCδ • PKCδ siRNA • PKCδ shRNA • PKCδ sgRNA (CRISPR/Cas9) • rottlerin* (i.p., 8 mg/kg; once per day for 12 weeks)	• APPswe/PS1dE9 transgenic mouse • SH-SY5Y-APP741 • Mouse primary cortical neurons (Tg2576) • N2a-APP695	• rottlerin mitigates neuritic plaque formation and rescues cognitive deficits in AD mice	• rottlerin reduces BACE1 expression and Aβ levels • PKCδ knockdown reduces BACE1 expression, BACE1-mediated APP processing, and Aβ production • PKCδ overexpression increases BACE1 expression and Aβ generation	[109]
• bryostatin-1* • benzolactams*	• SH-SY5Y cells	• sustained activation of PKCδ/ε associated with enhanced α-secretase processing	• bryostatin-1: non-selective activators of PKCδ/ε • benzolactams: activates PKC by directly binding to the regulatory C1 domain of PKCs	[128]
• nano-encapsulated bryostatin-1*	• APPswe/PS1dE9 transgenic mouse	• shorter latencies, increased % time in the target zone, and decreased % time in the opposite quadrant in Morris water maze test	• transient activation of PKCδ/ε	[129]
• EGFO* (oral, 50 or 200 mg/kg/day)	• ICV injection of Aβ1–42 into the C57BL/6 N mouse brains • H_2_O_2_-treated HT22 cells	• improved cognition based on passive avoidance task and Morris water maze test • increased hippocampal expression of NeuN • inhibited H_2_O_2_ toxicity in HT22 cells	• decreased phospho-PKCδ at Thr505	[130]

Aβ, amyloid-beta peptide; APP, amyloid precursor protein; BACE1, beta-site APP cleavage enzyme-1; bryostatin-1, a natural macrocyclic lactone isolated from *Bugula neritina*; benzolactams, small molecules; CDK5, cyclin-dependent kinase-5; CRISPR-Cas9, clustered regularly interspaced short palindromic repeats/CRISPR-associated protein 9; EGFO, *Elaeagnus glabra* f. *oxyphylla* extract; HT22, a mouse hippocampal cell line; ICV, intra-cerebroventricular; i.p., intraperitoneal; N2a, mouse neuroblastoma; NeuN, neuronal nuclei; PUMA, p53-upregulated modulator of apoptosis; sgRNA, single-guide RNA; SH-SY5Y, a human neuroblastoma cell line; STAT3, signal transducer and activator of transcription-3; *: non-specific for PKCδ

A separate issue concerns non-selective PKC activation. Beneficial effects reported with bryostatin-1, benzolactams, or nano-encapsulated bryostatin-1 do not necessarily indicate that PKCδ activation is therapeutic, because these compounds engage PKCα/PKCε-associated α-secretase pathways and may remodel multiple PKC isoforms [128, 129]. In light of the APP-processing literature discussed above, their cognitive or amyloid-related benefits are more plausibly attributed to isoform-mixed network effects, especially enhancement of non-amyloidogenic α-secretase processing by PKCα/PKCε, while any concurrent PKCδ activation may be neutral, context-dependent, or potentially deleterious. EGFO, an ethanol extract of *Elaeagnus glabra* f. *oxyphylla*, likewise illustrates pathway-level modulation rather than target validation: although its protective effects were associated with reduced PKCδ Thr505 phosphorylation in Aβ1-42-injected mice and H_2_O_2_-treated HT22 cells, it is a multi-component extract and PKCδ can only be considered one candidate mediator [130]. The cognitive and APP-processing benefits reported with broad PKC activators may therefore reflect, at least in part, activation of PKCα/PKCε-associated non-amyloidogenic α-secretase pathways, which could offset or obscure potentially deleterious PKCδ-dependent amyloidogenic, inflammatory, or neuronal stress responses. Thus, the apparent conflict between PKCδ-linked pathogenic studies and beneficial effects of broad PKC activators is best explained by isoform selectivity, cellular context, and network-level PKC remodeling. Future interventions should therefore distinguish PKCδ-specific inhibition from broader PKC-network modulation, particularly to avoid disrupting potentially beneficial PKCα/PKCε-associated non-amyloidogenic signaling.

### Critical Perspective and Future Directions for AD

Taken together, the AD literature supports a revised, but still cautious, view of PKCδ. Human postmortem evidence remains limited and inconsistent, preventing a simple conclusion that PKCδ is uniformly increased, causal, or detrimental in AD. Nevertheless, experimental data place PKCδ at three interconnected pathological nodes: (i) BACE1-associated amyloidogenic APP processing; (ii) Aβ-induced neuronal stress, cell cycle reentry, and apoptotic signaling; and (iii) astrocytic and microglial inflammatory amplification. Viewed as a cell-type-specific feed-forward amplifier, PKCδ provides a coherent mechanistic link between amyloidogenesis, neuronal injury, and neuroinflammation rather than independently modulating three unrelated pathways.

Several issues remain important to be considered. First, the neuronal injury pathway is supported most directly by in vitro studies in Aβ-treated neuronal systems, and additional in vivo evidence is needed. Second, the amyloidogenic APP-processing evidence is strengthened by in vivo APPswe/PS1dE9 data, but still requires more isoform-selective validation. Finally, broad PKC activator studies cannot be used as evidence for beneficial PKCδ activation because PKCα/PKCε-dependent α-secretase signaling likely contributes substantially to those effects. Future studies should therefore prioritize cell type-specific human analyses, activation-state and localization measurements, and PKCδ-selective interventions capable of distinguishing pathogenic PKCδ suppression from potentially beneficial PKCα/PKCε-associated non-amyloidogenic signaling. Such studies will determine whether PKCδ is best regarded as a biomarker of AD-related stress, a pathogenic amplifier, or a tractable therapeutic target.

## Huntington’s Disease

HD is an autosomal dominant neurodegenerative disorder caused by expansion of a CAG repeat in the huntingtin gene. Mutant huntingtin (mHtt) promotes selective striatal and cortical neurodegeneration through several interacting mechanisms, including mitochondrial dysfunction, oxidative stress, excitotoxic Ca^2+^ signaling, impaired proteostasis, transcriptional dysregulation, and apoptosis [131]. Although direct evidence linking PKCδ to HD remains relatively sparse compared with PD, AD, or ischemic stroke, the available studies suggest that PKCδ may function as a context-dependent stress-threshold modulator rather than as a uniformly detrimental or protective kinase. The major HD-related PKCδ studies, including their experimental models, proposed mechanisms, and inferred functional direction, are summarized in Table 3.

**Table 3:** Effects of pharmacological and experimental manipulation of PKCδ in HD pathology

Compound /Tool	Study Model	Downstream Effect	Mechanism	Ref.
• HA-tagged overexpression of PKCδ plasmid • dominant-negative PKCδK376R mutant • PKCδ siRNA	• HD transgenic mice R6/1 and R6/2 • HdhQ111/Q111 knock-in mice • postmortem HD brains • STHdhQ7/Q7 cells transfected with N-WT-Htt-CFP or N-mHtt-CFP	• Functional interpretation: context-dependent; reduced PKCδ abundance may be adaptive/protective, whereas PKCδ overexpression is detrimental • reduced PKCδ protein levels in the striatum and cortex of R6/2 and HdhQ111/Q111 knock-in mice, and in the putamen of HD patients	• increased phospho-PKCδ at Thr505 in R6/1 mouse brain at late stages • overexpression of PKCδ, but not a dominant-negative mutant, promotes cell death in mHtt-expressing cells • lower PKCδ levels prevent mHtt-induced cell death • PKCδ promotes lamin-B degradation in mHtt-expressing cells	[132]
• cadmium chloride (CdCl_2_)	• heterozygous mHtt mouse striatal cells (STHdhQ7/Q111)	• Functional interpretation: detrimental under second-hit oxidative stress • mHtt/Cd increased cell death via caspase mediated apoptosis, altered metal transport, and modulation of ERK- and PKCδ-dependent oxidative signaling mechanisms	• mHtt/Cd increased PKCδ expression and other key oxidative stress proteins • enhanced apoptosis mediated by caspase-9 and caspase-3 • blocked ERK expression • intracellular accumulation of Cd and reduced expression of DMT1 protein	[133]
• PKCδ RNAi • rottlerin*	• HdhQ111 knock-in mice • SH-SY5Y cells were transfected with human htt(1–171)150Q	• Functional interpretation: detrimental/pro-apoptotic • PKCδ partially contributed to mHtt-dependent Ser46 phosphorylation on p53 • Knockdown of PKCδ by RNAi decreased phospho-Ser46 on p53 and mHtt-dependent apoptosis	• mHtt enhanced the activities of ATM, HIPK2, and PKCδ, causing phosphorylation of p53 on Ser46 • Prolyl isomerization of the phospho–Ser46-Pro47 site by Pin1 unlocked p53 from the apoptosis inhibitor iASPP to induce apoptotic genes	[40]

ATM, ataxia telangiectasia mutated; Cd, cadmium; CFP, cyan fluorescent protein; DMT1, divalent metal transporter-1; ERK, extracellular signal-regulated kinase; HA, hemagglutinin; HD, Huntington’s disease; HIPK2, homeodomain-interacting protein kinase-2; Htt, huntingtin; iASPP, inhibitor of apoptosis-stimulating protein of p53; mHtt, mutant huntingtin; RNAi, RNA interference; WT-Htt, wild-type huntingtin. *: non-specific for PKCδ

### Context-Dependent Regulation of PKCδ in HD

Available evidence suggests that the early reduction of PKCδ observed in HD models and patient tissue may have biological significance beyond a simple loss of kinase expression. In R6/2 and Hdh(Q111/Q111) mice, PKCδ protein levels are reduced in the striatum and cortex, and decreased PKCδ expression has also been reported in the putamen of patients with HD. In parallel, phosphorylation and ubiquitination of PKCδ are increased in the brains of 30-week-old R6/1 mice. Functionally, PKCδ overexpression enhances mHtt-induced cell death, whereas dominant-negative PKCδ expression or knockdown of endogenous PKCδ partially attenuates mHtt toxicity [132]. While more experimental evidence is needed, these observations nevertheless suggest the possibility that early PKCδ downregulation may represent a compensatory proteostatic response, which limits the availability of a pro-apoptotic kinase to delay the transition from chronic mHtt-associated cellular stress to overt neuronal death.

This interpretation is also consistent with the broader concept that HD neurons undergo prolonged sublethal stress before frank degeneration becomes evident. In such a setting, reduced PKCδ abundance may help raise the threshold for apoptotic execution by restraining PKCδ-dependent caspase amplification, nuclear apoptotic signaling, or stress-responsive kinase signaling. However, this apparent adaptation should not be interpreted as evidence that PKCδ is irrelevant to HD progression. Rather, the same pathway may become pathogenic when compensatory suppression is insufficient or when additional stressors reactivate PKCδ-linked death signaling.

### Potential Intersections Between PKCδ and Canonical HD Pathogenic Mechanisms

This stage-dependent model provides a framework for integrating PKCδ with established HD-associated mechanisms. Mitochondrial dysfunction, impaired oxidative phosphorylation, defective Ca^2+^ handling, and oxidative stress are closely interconnected in HD. Although the available HD-specific PKCδ literature does not yet demonstrate a direct causal role for PKCδ in mitochondrial failure or excitotoxicity, PKCδ is positioned near several downstream consequences of these insults, including oxidative stress responses, caspase activation, and apoptosis. Thus, early PKCδ suppression may be interpreted as an adaptive attempt to buffer neurons against the pro-apoptotic consequences of chronic mitochondrial and excitotoxic stress, rather than as an isolated change in kinase expression.

Proteostatic dysfunction may represent another point of convergence. The observation that PKCδ phosphorylation and ubiquitination are increased in HD mouse brain suggests that PKCδ abundance may be regulated through post-translational modification and protein turnover during disease progression [132]. Because HD is characterized by impaired protein quality control, aggregation of mHtt, as well as disturbances in ubiquitin-proteasome and autophagy-related pathways, regulated PKCδ ubiquitination may reflect an attempt to remove or restrain a pro-death kinase under chronic mHtt stress. This interpretation remains inferential, but it provides a mechanistic explanation for why reduced PKCδ expression during early disease stages may be biologically meaningful rather than merely epiphenomenal.

A more direct mechanistic link between PKCδ and mHtt toxicity is provided by the p53 apoptotic pathway. Grison et al. showed that mHtt promotes phosphorylation of p53 at Ser46, enabling Pin1-dependent conformational regulation of p53, dissociation of p53 from the inhibitor of apoptosis-stimulating protein of p53 (iASPP), and induction of apoptotic target genes. In this pathway, PKCδ together with ataxia telangiectasia mutated (ATM) and homeodomain-interacting protein kinase-2 (HIPK2), was implicated as an upstream stress-responsive kinase contributing to p53 Ser46 phosphorylation; pharmacological inhibition of PKCδ with rottlerin, as well as PKCδ knockdown, reduced mHtt-induced Ser46 phosphorylation on p53 and apoptosis [40]. This finding connects PKCδ not only to apoptotic signaling in HD but also to a specific p53-dependent transcriptional death program. Therefore, PKCδ may intersect with transcriptional dysregulation in HD most directly through stress-induced activation of apoptotic gene expression, rather than through broad regulation of all mHtt-associated transcriptional abnormalities.

A complementary stress-amplification model is supported by studies of cadmium exposure in heterozygous mHtt striatal cells. In this setting, mHtt expression increases vulnerability to cadmium-induced cytotoxicity, which is associated with NOX-dependent oxidative stress, increased PKCδ expression, enhanced caspase-9 and caspase-3 activation, and apoptosis. The protective effects of antioxidants and apocynin, the inhibitor of NOX, further suggest that oxidative stress can function as a second hit that unmasks PKCδ-associated apoptotic signaling in mHtt-expressing striatal cells [133]. Although cadmium exposure should not be regarded as a core HD mechanism, this model illustrates how environmental or metabolic stressors may shift PKCδ from an adaptively suppressed state toward a pro-apoptotic signaling node.

Taken together, these observations support a stage- and stress-dependent model of PKCδ function in HD. During early disease stages, reduced PKCδ abundance may represent an adaptive attempt to restrain PKCδ-dependent apoptosis and preserve neuronal viability. As mitochondrial dysfunction, oxidative stress, excitotoxic Ca^2+^ stress, impaired protein quality control, and transcriptional dysregulation accumulate, this compensatory suppression may become insufficient. Under these conditions, PKCδ may participate in stress-responsive apoptotic signaling through pathways such as p53 Ser46 phosphorylation and caspase activation. This dual role helps explain why PKCδ in HD should be discussed as a context-dependent modulator of neuronal vulnerability rather than as a simple linear therapeutic target.

### Therapeutic Implications and Limitations of PKCδ Modulation in HD

At present, no pharmacological agents specifically targeting PKCδ have been established as HD therapeutics in either preclinical animal studies or clinical trials. Likewise, there are no published gene-therapy approaches, such as adeno-associated virus (AAV)- or clustered regularly interspaced short palindromic repeats (CRISPR)-based strategies, designed specifically to modulate PKCδ expression or activity in HD models. Although rottlerin has been widely used in basic research as a putative PKCδ inhibitor, its selectivity is controversial as discussed above in this review, and its effects in HD-related cellular systems should therefore be interpreted cautiously. Nevertheless, experimental manipulation of PKCδ expression, together with stress paradigms such as cadmium exposure, indicates that PKCδ can influence mHtt-associated striatal vulnerability under specific cellular contexts.

These findings suggest that indiscriminate PKCδ inhibition is unlikely to be a straightforward therapeutic strategy for HD. If PKCδ is already downregulated during early disease stages as an adaptive response, further suppression may not provide additional benefit and could theoretically interfere with context-specific kinase functions. Conversely, targeting stress-induced PKCδ reactivation may be more relevant under conditions in which mitochondrial dysfunction, oxidative stress, metal dyshomeostasis, DNA-damage signaling, or p53-dependent apoptotic transcription become dominant. Any future PKCδ-directed intervention in HD would therefore require careful attention to disease stage, cell type, subcellular PKCδ localization, and trigger-specific signaling context.

## Ischemic Stroke

Ischemic stroke is a major cause of death and long-term disability worldwide. Tissue injury is initiated by the loss of cerebral blood flow, but restoration of perfusion can itself amplify damage through a burst of ROS with resultant oxidative stress, leukocyte recruitment, BBB disruption, mitochondrial failure, and programmed cell death. Within this setting, PKCδ has emerged as one of the more consistently injury-associated PKC isoforms in experimental stroke. The available literature supports more than a simple role for PKCδ in the ischemic core alone; rather, current evidence places PKCδ at the interface between reperfusion, inflammatory cell activation, oxidant production, and secondary expansion of tissue injury. This distinction is important, because it helps explain why PKCδ inhibition tends to be most effective in transient ischemia or oxygen-glucose deprivation/reoxygenation (OGD/R) paradigms rather than in permanent occlusion models [134, 135].

### Direct PKCδ-Manipulation Evidence in Reperfusion-Associated Injury

To distinguish causality from association, the ischemic stroke literature can be separated into direct PKCδ-manipulation studies and indirect pathway-modulating interventions. The most compelling evidence that PKCδ is detrimental in ischemic stroke comes from genetic and pharmacological studies focusing on reperfusion injury. In transient middle cerebral artery occlusion (MCAO) with reperfusion (MCAO/R), PKCδ-null mice develop substantially smaller infarcts and show improved neurological outcomes compared with wild-type controls, whereas this protection is not evident after permanent ischemia without reperfusion [136]. This pattern argues that PKCδ contributes primarily to reperfusion-dependent injury rather than to the immediate consequences of blood flow cessation per se. Bone marrow transplantation experiments further refined this conclusion by showing that PKCδ in hematopoietic cells, especially neutrophils, is a major determinant of injury severity. Wild-type recipients reconstituted with PKCδ-deficient bone marrow are protected, whereas PKCδ-null recipients receiving wild-type marrow lose much of that protection, supporting the concept that neutrophil PKCδ constitutes a substantial component of post-ischemic inflammatory damage [46].

Selective pharmacological interference with PKCδ translocation provides a second line of direct evidence. The PKCδ-selective inhibitory peptide δV1-1, also reported as transactivator of transcription (TAT)-δV1-1 when fused to a cell-penetrating TAT sequence, reduces tissue injury in organotypic hippocampal slice models and in vivo cerebral ischemia-reperfusion paradigms when present during ischemia and early reperfusion. These findings indicate that PKCδ activation and translocation are not merely epiphenomena of dying tissue but participate functionally in injury propagation. Mechanistically, these direct-manipulation studies support a model in which PKCδ promotes reperfusion damage through inflammatory amplification, pro-death signaling, and compartment-specific translocation events that become especially relevant when oxygen delivery is restored [136–139].

### Indirect Pathway-Modulating Evidence Linking PKCδ to Oxidative, Vascular, and Mitochondrial Injury

A broader and more indirect body of literature links PKCδ to oxidative stress, Akt-associated survival signaling, vascular injury, edema, mitochondrial dysfunction, and cell death in ischemic stroke. Because reperfusion rapidly increases ROS, it is plausible that PKCδ both responds to and further amplifies oxidative injury. Several intervention studies converge on this idea, although many should be interpreted as supportive rather than strictly causative because these interventions also alter multiple pathways in parallel besides PKCδ. Chlorpromazine plus promethazine treatment, for example, reduces infarct burden while decreasing PKCδ expression and increasing Akt phosphorylation in post-ischemic brain [140]. Follow-up work examining the NOX-Akt-PKCδ axis reported that this regimen suppresses NOX activity and lowers PKCδ, although the causal ordering of NOX, Akt, and PKCδ was not fully resolved; notably, the NOX inhibitor apocynin further reduced PKCδ without augmenting Akt phosphorylation, suggesting that PKCδ regulation may only be partially coupled to Akt in this context [141]. Related findings were obtained in thromboembolic stroke models treated with normobaric oxygen combined with therapeutic hypothermia or ethanol, where neuroprotection was accompanied by attenuation of a PKCδ-Akt-NOX signaling program following administration of recombinant tissue plasminogen activator (rt-PA) [142]. Ethanol pretreatment before reperfusion also decreases PKCδ expression while increasing total Akt abundance and reducing cell death [143]. Thus, Akt-linked changes are best viewed as part of a broader protective signaling signature rather than as definitive evidence that PKCδ is uniformly upstream or downstream of Akt in all stroke paradigms.

Among intervention-based studies, the most direct mechanistic support for a link between PKCδ and oxidative stress comes from the work using both MCAO/R in rats and OGD/R in SH-SY5Y cells. In that study, chlorpromazine plus promethazine lowered ROS production, reduced NOX subunit expression and activity, suppressed PKCδ phosphorylation, diminished PKCδ membrane translocation, and weakened the interaction between PKCδ and p47phox. These data more convincingly position PKCδ as an active participant in NOX-centered oxidant signaling, rather than just a passive downstream marker of cellular stress [45]. Overall, current evidence supports the view that PKCδ contributes to oxidative injury in ischemic stroke, but the relative contribution of PKCδ from different cell systems, namely neuronal, vascular, or leukocyte, in driving ROS remains insufficiently resolved.

Indirect pathway-modulating evidence also links PKCδ to edema formation, BBB-associated injury, mitochondrial dysfunction, and apoptotic signaling. Thyroxine treatment after ischemia reduces infarct size while lowering phosphorylated PKCδ and simultaneously enhancing expression of anti-apoptotic, anti-inflammatory, and neurotrophic genes [144]. Although PKCδ was not manipulated directly in that model, the supportive evidence from this study broadens the range of injury programs that may intersect with PKCδ beyond the canonical oxidative stress pathway. Particularly notable is the work linking PKCδ to matrix metalloproteinase-9 (MMP9), aquaporin-4 (AQP4), and cerebral edema. In focal ischemia, upregulation of PKCδ correlates with increased MMP9 activity and enhanced AQP4-associated water entry, changes expected to worsen vasogenic and cytotoxic swelling. Intra-arterial mesenchymal stem cell therapy attenuates these responses while reducing PKCδ expression, suggesting that suppression of PKCδ may contribute to protection of the BBB and limitation of edema progression [145]. More recent work from the same group linked this effect to increased sirtuin-1 (SIRT1) signaling, reduced NF-κB activity, lower ROS burden, and improved mitochondrial integrity, again with reduced PKCδ expression as part of the protective signature [146].

Mitochondrial dynamics provide another plausible effector arm. YiQiFuMai treatment ameliorates ischemic injury in vivo and H_2_O_2_-induced damage in primary cortical neurons by suppressing PKCδ/dynamin-related protein-1 (Drp1)-dependent excessive mitochondrial fission, restoring MMP and improving ATP production, as well as reducing apoptosis [39]. These findings are mechanistically attractive because PKCδ is well positioned to integrate oxidative stress with mitochondrial fragmentation and death execution. Nevertheless, because YiQiFuMai is a multi-component intervention, this evidence should be interpreted as pathway-supportive rather than as definitive proof that PKCδ alone is the causal driver of mitochondrial fission in ischemic injury.

### Therapeutic Implications and Translational Limitations of PKCδ Modulation

Experimental strategies targeting or modulating PKCδ in ischemic stroke, including genetic deletion, peptide-based translocation blockade, and pleiotropic pathway-modulating interventions, are summarized in Table 4. The direct genetic and peptide-inhibition evidence described above supports the concept that PKCδ modulation may be therapeutically relevant in cerebral ischemia-reperfusion injury. Among available approaches, the TAT-conjugated δV1-1 (TAT-δV1-1) remains the best-characterized PKCδ-targeting peptide in experimental stroke models [136, 147]. TAT-δV1-1 is derived from the V1 region of PKCδ (SFNSYELGSL) and selectively disrupts PKCδ translocation rather than directly inhibiting catalytic activity. The δV1-1 sequence mimics the receptor for activated C kinase (RACK)-binding site within the regulatory domain of PKCδ and competitively blocks interaction with the isozyme-specific anchoring protein δRACK. By preventing stimulus-induced anchoring of activated PKCδ to subcellular signaling compartments, particularly mitochondrial or particulate membrane compartments in ischemia-reperfusion models, TAT-δV1-1 inhibits spatially restricted phosphorylation of downstream substrates and attenuates pro-apoptotic signaling during ischemic injury [136, 147].

**Table 4:** Pharmacological and experimental strategies targeting PKCδ in ischemic stroke

Compound /Tool	Study Model	Downstream Effect	Mechanism	Ref.
• PKCδ^−/−^ mice	• pMCAO • tMCAO	• reduced infarct size in tMCAO, but not in pMCAO • attenuated neutrophil function in PKCδ^−/−^ mice	• PKCδ-mediated neutrophil adhesion, migration, degranulation, and superoxide generation	[46]
• TAT-δV1-1	• OGD/R in rat hippocampal slice • MCAO/R in rats	• reduced cellular injury • decreased infarct size	• ↓ TUNEL+ cells • ↑ p-Akt • ↓ translocation of BAD from cytosol to membrane	[136]
• TAT-δV1-1	• MCAO/R in normotensive rats • chronic hypertensive models • MCAO/R in the Dahl salt-sensitive hypertensive rats	• improved microvascular pathology in normotensive rats with MCAO/R • increased cerebral blood flow in chronic hypertension model • reduced infarct size in hypertensive rats	• increased the number of patent microvessels • mitigated microvascular damages (e.g., vascular edema, swollen astrocyte end-foot processes, basal lamina breakdown, and compression of vascular lumen)	[137]
• TAT-δV1-1	• MCAO/R with RPostC	• RPostC effects: • improved neurologic outcome • reduced infarct size • inhibited neuronal apoptosis • suppressed PKCδ activation	• conferred neuroprotection against cerebral reperfusion injury	[138]
• TAT-δV1-1	• global ischemia with reperfusion • two-vessel occlusion/ hypotension in rats • ACA in rats	• attenuated hyperemia and hypoperfusion/ vasoconstriction followed by vasodilation of microvessels in two-vessel/ hypotension • decreased hippocampal CA1 and cortical neuronal death 7 days after ACA	• prevented neuronal injury by modulation of CBF	[139]
• C + P*	• pMCAO • tMCAO	• reduced infarct volumes and neurological deficits in both tMCAO and pMCAO	• ↑ p-Akt • ↓ PKCδ • ↓ ROS • ↑ ATP • ↑ NADH activity	[140]
• C + P*	• tMCAO	• reduced infarct volumes and neurological deficits	• ↓ PKCδ • ↓ Bax • ↑ Bcl-xL • ↓ caspase-3 • ↓ apoptosis	[141]
• C + P*	• tMCAO • SH-SY5Y subjected to OGD/R	• reduced infarct volumes and neurological deficits	• ↓ PKCδ/pPKCδ • ↓ NOX • ↑ MnSOD • ↓ ROS • ↓ apoptosis	[45]

ACA, asphyxial cardiac arrest; BAD, bcl-2-associated death protein; Bax, Bcl-2-associated X protein; Bcl-xL: B-cell lymphoma-extra-large. C + P: chlorpromazine and promethazine; CBF, cerebral blood flow; MCAO/R, middle cerebral artery occlusion with reperfusion; MnSOD, manganese superoxide dismutase; NADH, nicotinamide adenine dinucleotide in its reduced form; NOX, nicotinamide adenine dinucleotide phosphate oxidase; OGD/R, oxygen-glucose deprivation with re-oxygenation; p-Akt, phosphorylated Akt; pMCAO, permanent MCAO without reperfusion; pPKCδ, phosphorylated PKCδ; ROS, reactive oxygen species; RPostC, remote ischemic post-conditioning; SH-SY5Y, human neuroblastoma cell line; tMCAO, transient MCAO with reperfusion; TUNEL, terminal deoxynucleotidyl transferase dUTP nick-end labeling. *: non-specific for PKCδ

In rat hippocampal slices subjected to OGD and in rat MCAO models, TAT-δV1-1 reduced neuronal injury, decreased infarct volume, limited the extent of TUNEL staining indicative of apoptosis, increased Akt phosphorylation, and prevented BAD translocation to mitochondria, thereby suppressing downstream apoptotic cascades [136]. Later work further showed that systemic administration at reperfusion improves neurological outcomes and is mechanistically linked to redox-sensitive suppression of PKCδ signaling [138]. Additional studies indicate that TAT-δV1-1 can improve post-ischemic cerebral blood flow, attenuate reactive hyperemia, delay hypoperfusion, and preserve microvascular structure and function [137, 139]. Taken together, these data provide relatively strong preclinical support for PKCδ translocation blockade as a potential therapeutic strategy in experimental ischemic stroke, but they should be interpreted primarily as proof-of-mechanism evidence rather than evidence of established clinical feasibility.

The major translational challenge for δV1-1-type inhibitors is delivery into CNS. Because the native δV1-1 peptide has poor cell permeability, it is typically fused to the human immunodeficiency virus type-1 (HIV-1) TAT sequence (YGRKKRRQRRR; amino acids 47–57) to enhance intracellular delivery and, in some rodent stroke studies, to facilitate access to brain parenchyma. However, efficient TAT-mediated uptake by endothelial or neural cells does not equate with predictable or therapeutically sufficient BBB penetration. Studies of other TAT-conjugated neuroprotective peptides have shown that TAT can promote robust internalization into BBB endothelial cells while producing much less efficient permeation across in vitro BBB models and the extent of BBB permeation in healthy rats is low [148]. Cargo conjugation to TAT can also alter BBB adherence, endothelial uptake, transcytosis, and chemical stability [149]. More broadly, the in vivo behavior of cell-penetrating peptides (CPPs) like TAT is highly cargo- and context-dependent: CPPs may show nonspecific tissue uptake, limited brain accumulation, high uptake in liver and kidney, rapid blood clearance, serum/proteolytic instability, and variable endosomal escape [150, 151]. Therefore, although the TAT moiety improves experimental intracellular delivery, it does not by itself resolve the key pharmacological questions relevant to clinical CNS application, including quantitative brain exposure, regional distribution within injured and non-injured brain, duration of PKCδ target engagement, reproducibility of delivery across intact, partially disrupted, or reconstituting BBB, and potential off-target systemic accumulation.

KAI-9803 (delcasertib) provides a useful translational cautionary example. It is a clinically developed PKCδ V1 region-derived, TAT47-57-conjugated peptide inhibitor that disrupts PKCδ translocation. In cardiac ischemia-reperfusion settings, KAI-9803 showed early promise in preclinical studies and in a first-in-human intracoronary study during primary percutaneous coronary intervention [152]. Pharmacokinetic and tissue-distribution work further demonstrated rapid TAT47-57-mediated delivery into target cells in liver, kidney, and heart after intravenous bolus administration [153]. These findings support the feasibility of systemic delivery of a δV1-1-derived peptide, but they also highlight broad peripheral distribution and do not establish adequate CNS exposure, BBB penetration, brain pharmacokinetics, or sustained PKCδ target engagement in human neurological disease. Moreover, the larger PROTECTION AMI phase II trial in patients with acute ST-segment elevation myocardial infarction undergoing primary percutaneous coronary intervention found that intravenous delcasertib did not reduce biomarker-defined infarct size, improve ST-segment recovery, improve left ventricular ejection fraction, or reduce adjudicated clinical events [154]. Thus, δV1-1/KAI-9803 should be discussed as important proof-of-concept strategies for blocking PKCδ translocation rather than as clinically validated CNS therapeutics. Future development will likely require delivery systems or next-generation PKCδ modulators with improved isoform selectivity, BBB transport, proteolytic stability, pharmacokinetic/pharmacodynamic characterization, as well as clinically practical routes and dosing regimens.

### Critical Appraisal and Future Directions for Ischemic Stroke

Accordingly, direct evidence in ischemic stroke is derived primarily from PKCδ-null mice, bone marrow transplantation, and δV1-1/TAT-δV1-1-mediated blockade of PKCδ translocation. In contrast, studies using chlorpromazine/promethazine, ethanol, therapeutic hypothermia, thyroxine, mesenchymal stem cell therapy, or YiQiFuMai should be interpreted as indirect or supportive because these interventions, in addition to PKCδ, alter multiple pathways in parallel. This evidence hierarchy is important for avoiding overinterpretation of associative pathway changes as direct PKCδ causality.

Taken together, the literature in ischemic stroke supports a more substantial pathogenic role of PKCδ as compared to some rare neurodegenerative disorders. The strongest claims are that PKCδ is predominantly deleterious in transient ischemia/reperfusion, that hematopoietic-neutrophil PKCδ is a major contributor to injury, and that selective interference with PKCδ signaling can reduce infarct size and improve functional outcome in experimental systems. A second, moderately supported layer of evidence links PKCδ to NOX-dependent oxidative stress, mitochondrial injury, edema-related signaling, and apoptotic execution. However, given that many interventions altering PKCδ also modulate numerous other pathways, it is often difficult to assign a causal relationship exclusively to PKCδ.

Therefore, at present PKCδ may be considered a reperfusion-amplifying kinase rather than a universal mediator of all ischemic injury. Its actions appear to operate in a multicellular network involving neutrophils, vascular elements, and injured neural tissue, with oxidative stress and mitochondrial dysfunction serving as recurring mechanistic bridges. Important unresolved questions remain to be answered. These include whether neuronal and myeloid PKCδ play separable temporal roles, whether PKCδ directly controls BBB failure in a cell-autonomous manner, and whether delayed inhibition after reperfusion onset retains a clinically meaningful therapeutic window.

## Cell Type-Specific and Context-Dependent Roles of PKCδ Across Neurological Disorders

Although PKCδ has often been discussed within individual disease categories, its biological effects in the CNS are strongly shaped by cellular context. Similar upstream stress signals, including oxidative stress, inflammatory cytokines, proteotoxic aggregates, and ischemia/reperfusion, may produce divergent outcomes depending on whether PKCδ is activated in neurons, microglia, astrocytes, or infiltrating immune cells. Thus, a cell-specific perspective helps integrate disease-specific findings and clarifies why PKCδ signaling may be protective, compensatory, or detrimental depending on the timing, intensity, and cellular compartment of activation. In several paradigms, transient PKCδ activation may initially represent a stress-responsive or compensatory signal, whereas sustained, excessive, or spatially misdirected PKCδ signaling is more likely to amplify neuronal injury, glial inflammation, or neurovascular damage.

In neurons, PKCδ is most consistently linked to mitochondrial dysfunction, aberrant stress signaling, and apoptotic cell death. In dopaminergic models relevant to PD, oxidative and neurotoxic insults can induce PKCδ activation, including caspase-3-dependent proteolytic cleavage, thereby amplifying apoptotic signaling and dopaminergic neuronal loss [74]. In AD-related models, Aβ exposure has been reported to induce PKCδ-dependent cell-cycle re-entry and apoptosis in post-mitotic cortical neurons, involving upstream STAT3 signaling and downstream calpain/p25/CDK5 and PUMA/caspase-3 pathways [117]; reciprocally, PKCδ also enhances Aβ production via promotion of β-secretase-associated APP processing [109], thereby supporting a self-amplifying vicious model connecting PKCδ, Aβ generation, and downstream neuronal stress including mitochondrial and apoptotic injury. In the context of HD, however, increased neuronal PKCδ phosphorylation, cleavage, expression, or subcellular redistribution should not automatically be interpreted as evidence that PKCδ is the initiating cause of neuronal degeneration; instead, it may reflect a downstream stress marker or a failed compensatory response.

In microglia, PKCδ appears to function primarily as an amplifier of inflammatory activation rather than as a direct apoptotic mediator. In experimental PD models, inflammatory and disease-relevant stimuli such as LPS, TNF-α, and aggregated α-synuclein increase microglial PKCδ expression, kinase activity, and nuclear translocation, leading to enhanced NF-κB signaling and production of pro-inflammatory mediators [47]. More recent AD-related evidence indicates that PKCδ is elevated in the cerebrospinal fluid of AD patients, is expressed predominantly in microglia in brain tissue, and contributes to Aβ-induced NF-κB activation and cytokine production; downregulation or inhibition of PKCδ attenuates these microglial responses and improves cognitive outcomes in an AD mouse model [54]. These observations suggest that chronic or excessive microglial PKCδ activation may sustain neuroinflammation across neurodegenerative disorders. However, because microglia can also contribute to debris clearance, trophic support, and tissue repair, the pathological significance of PKCδ activation is likely to depend on disease stage, duration of activation, and the balance between protective and detrimental microglial functions.

Astrocytic PKCδ has been less extensively characterized, but available studies suggest that it may link mitochondrial oxidative stress to reactive astroglial inflammation. In MPTP-based PD models, TWEAK/fibroblast growth factor-inducible 14 (Fn14) signaling has been shown to activate PKCδ in astrocytes and is associated with mitochondrial oxidative stress, STAT3 activation, NOD-like receptor family CARD domain-containing protein 4 (NLRC4) inflammasome signaling, and increased release of inflammatory cytokines, including IL-1β, TNF-α, and IL-18; PKCδ knockdown or mitochondrial antioxidant intervention suppresses these astrocytic inflammatory responses [51]. Given the central roles of astrocytes in glutamate buffering, metabolic support, maintenance of BBB integrity, and neuron-glia communication, astrocytic PKCδ may influence neuronal vulnerability indirectly even when the primary site of PKCδ activation is not neuronal. At the same time, the astrocytic literature remains comparatively limited, and the relevance of astrocytic PKCδ beyond specific PD-related inflammatory paradigms should be interpreted with caution.

In infiltrating immune cells, the clearest evidence for PKCδ involvement comes from ischemic stroke and reperfusion injury. PKCδ-null mice exhibit reduced injury after transient, but not necessarily permanent, MCAO. This protection is associated with impaired neutrophil adhesion, migration, respiratory burst, degranulation, and reduced neutrophil infiltration into ischemic tissue; bone marrow transplantation experiments further support a hematopoietic/neutrophil contribution to PKCδ-dependent reperfusion injury [46]. Thus, in acute vascular injury, PKCδ appears to promote neurovascular inflammation by facilitating leukocyte recruitment and effector functions. This mechanism is particularly relevant to reperfusion-associated damage and should not be broadly extrapolated to all chronic neurodegenerative disorders. Moreover, because peripheral immune cells may also participate in host defense, debris clearance, and tissue remodeling, broad systemic inhibition of PKCδ could have context-dependent consequences.

Taken together, these observations indicate that PKCδ should be viewed as a context-dependent stress-responsive kinase rather than a uniformly deleterious molecule. Neuronal PKCδ is most often associated with mitochondrial and apoptotic injury; microglial PKCδ with inflammatory amplification; astrocytic PKCδ with reactive gliosis and inflammasome-related responses; and neutrophil/leukocyte PKCδ with acute reperfusion-associated neurovascular inflammation. This cell-specific framework also has therapeutic implications. Non-selective PKCδ inhibition may suppress convergent injury pathways in selected disease settings, but optimal translation will likely require attention to disease stage, duration of activation, target cell population, CNS penetration, and the need to preserve adaptive stress responses or reparative immune and glial functions.

## Overall Conclusions and Future Perspectives

Across the neurological disorders discussed in this review, the translational strength of the PKCδ literature should be interpreted according to the nature of the supporting evidence. Genetic deletion, RNA interference, cleavage-resistant mutants, and peptide inhibitors provide strong mechanistic evidence that PKCδ can amplify oxidative, inflammatory, mitochondrial, and apoptotic injury in selected experimental settings. However, much of this evidence derives from cell culture systems, toxin-based models, acute injury paradigms, genetic manipulations, or nonselective pharmacological interventions. Direct human evidence remains relatively limited and often relies on postmortem tissue, total protein abundance, or pathway-associated observations rather than disease-stage-resolved measurements of PKCδ phosphorylation, proteolytic cleavage, kinase activity, or cell type-specific localization. Thus, PKCδ should currently be viewed as a disease-relevant and experimentally tractable signaling node, rather than an already validated clinical therapeutic target.

Taken together, the available literature identifies PKCδ as an important regulator of neuronal and glial stress responses in neurodegenerative disease and cerebral ischemia. In PD and ischemic stroke, the preclinical evidence for detrimental PKCδ signaling is comparatively strong, particularly in paradigms involving oxidative stress, mitochondrial dysfunction, neuroinflammation, and reperfusion-associated injury. In AD and HD, by contrast, PKCδ appears more context-dependent, with effects that vary according to disease stage, cell type, upstream stimulus, and interaction with other PKC isoforms. These distinctions argue against a uniform pathogenic interpretation of PKCδ across CNS disorders and emphasize the need to distinguish acute adaptive responses from chronic injury-amplifying signaling.

Future therapeutic development should therefore move beyond indiscriminate PKCδ inhibition toward isoform-selective and context-resolved modulation. The most promising strategies may include isoform-selective small-molecule inhibitors, peptide-based or allosteric approaches that block pathological PKCδ translocation or caspase-mediated cleavage, targeted delivery systems capable of achieving adequate CNS exposure, and upstream interventions that limit sustained oxidative or inflammatory activation of PKCδ. Thus, the therapeutic goal should not be global suppression of PKCδ activity, but selective interruption of maladaptive PKCδ signaling in the relevant disease stage, cell type, and subcellular compartment while preserving adaptive or reparative functions. In this respect, isoform-selective PKCδ targeting remains a compelling but still preclinical opportunity for neurodegenerative diseases and ischemic injury. Its future success will depend on disease-relevant models and human-derived systems that define when, where, and in which cells PKCδ becomes a pathological amplifier rather than a compensatory stress-response kinase.
